# A RCT for assessment of active human-centred learning finds teacher-centric non-human teaching of evolution optimal

**DOI:** 10.1038/s41539-020-00078-0

**Published:** 2020-12-04

**Authors:** Loredana Buchan, Momna Hejmadi, Liam Abrahams, Laurence D. Hurst

**Affiliations:** The Milner Centre for Evolution, Department of Biology and Biochemistry, University of Bath, Bath, BA2 7AY UK

**Keywords:** Education, Psychology

## Abstract

Current educational discourse holds that effective pedagogy requires engagement through active student participation with subject matter relating to them. The lack of testing of lessons in series is recognized as a potential weakness in the evidence base, not least because standard parallel designs cannot capture serial interaction effects (cf. drug interactions). However, logistic issues make large-scale replicated in situ assessment of serial designs challenging. The recent introduction of evolution into the UK primary school curriculum presents a rare opportunity to overcome this. We implemented a randomised control 2 × 2 design with four inexpensive schemes of work, comparable to drug interaction trials. This involved an initial test phase (*N* = 1152) with replication (*N* = 1505), delivered by teachers, after training, in their classrooms with quantitative before-after-retention testing. Utilising the “genetics-first” approach, the schemes comprised four lessons taught in the same order. Lessons 1 (variation) and 3 (deep-time) were invariant. Lesson 2 (selection) was either student-centred or teacher-centred, with subject organism constant, while lesson 4 (homology) was either human-centred or not, with learning mode constant. All four schemes were effective in replicate, even for lower ability students. Unexpectedly, the teacher-focused/non-human centred scheme was the most successful in both test and replicate, in part owing to a replicable interaction effect but also because it enabled engagement. These results highlight the importance of testing lessons in sequence and indicate that there are many routes to effective engagement with no “one-size fits all” solution in education.

## Introduction

Current educational discourse holds that actively involving students in the classroom, particularly using lessons that are more personally relevant (human centric) are vital for the process of learning^1^. In evolution education, the focus of this study, the use of humans and other primates to illustrate concepts of evolution has been shown to be important^2^. This assumes that the degree of student engagement through active learning is correlated with the likelihood of conceptual change^3^. These presumptions have influenced education policy in various countries, including the UK, which puts active learning at the heart of the curriculum^4^.

There is some experimental data to support this primacy given to the efficacy of active learning compared to traditional didactic or teacher-centred methods^5^ in helping undergraduates develop scientific understanding^6^. For school-age pupils it has been suggested that “hands on” lessons^7^ which are student-centred should be optimal^8^. The spectrum of practice can be diverse, ranging from inquiry-based lessons, case studies and modelling. Educational policy and practice around active and passive learning constructs tend to be based on dichotomous generalisations, despite the range of dimensions within these constructs which focus on the development of conceptual understanding. For the purposes of this study, we use the term active learning to represent student-centred, “hands-on” activities compared to teacher-centred, presentation-style content delivery^9^.

While the current educational theory is commonly accepted, the findings from these studies come with potentially acute caveats. First, relatively little school-age testing has been done (most studies are on University students), and what has been done is typically under controlled conditions without using teachers in situ. These conditions do not reflect realistic settings, and instruction given by academic experts fails to mimic the real-world context where teacher confidence in delivery is a potentially important variable.

Second, and perhaps more importantly, while teaching is done in series (one lesson after the other), tests of methodology are typically done in parallel (A v not A) with no regard to prior or subsequent teaching, for example, comparing active with passive learning but not considering how this might interact with prior or subsequent teaching. Implicitly we assume there to be no interaction effects. This is the educational equivalent of performing a drug trial but without considering drug interactions. Considering possible interactions, as with drug interaction tests, requires much larger sample sizes for the same degree of power. Nonetheless, iterative interactions between procedural and conceptual knowledge have been described in sequences of Mathematics lessons in primary school aged children^10^. In this context, Leach and Scott^11^ emphasised the dearth of studies comparing the effectiveness of sequences of lessons in achieving learning goals. Indeed, a limitation of all tests of primary school teaching of evolution (at least that we are aware of) is that they are limited to considering the effectiveness of singular stand-alone activities.

The need for large sample sizes, especially true for in-series testing, imposes considerable logistical demands as regards recruitment, retention and compliance. With busy teachers, retention and recruitment are acute issues, alongside teacher non-compliance of in situ testing of teaching packages, especially with specialist teachers delivering well-practiced lessons. Teaching by the researchers in a controlled setting avoids this problem but limits sample sizes, especially when evaluation is qualitative. Sample sizes thus tend to be small and replication rare, no matter what the approach.

Primary school teaching of evolution in the UK provides, we suggest, an exceptional and rare opportunity to short-circuit the above limitations. As evolution has only recently been introduced into the UK primary school curriculum (2014) this presents the possibility of an unusually “clean” in situ trial. Being largely non-specialist, primary school teachers are unusually receptive to the provision of resources and training, rendering recruitment to a randomised trial easier than it might otherwise have been. Further, and perhaps more importantly, with such teachers often lacking confidence in teaching evolution and with no prior well-developed teaching plans, adherence to prescribed series of lessons in teaching interventions is likely to be high. We emphasise that adherence to a series of lessons is most unusual. In drug trial terminology, we expect high recruitment, compliance and completion.

Here then, we consider the optimal mode of learning employing a sequential experimental design to allow for interaction effects, using primary school teaching as an exemplar. Evolution as a subject is also well suited to testing alternative teaching modes, it being relatively easy to design lessons to alter the above two axes. For example, we employ the peppered-moth exemplar and teach natural selection with pupils either as actively engaged birds pecking at moths or via a teacher-centred PowerPoint lesson. To teach the concept of homology and common ancestry, we have the material relate to the student or not. We employ the evolutionary history of the bones of their limbs as the human-centred lesson and employ trilobites as the non-human alternative. Trilobites were considered appropriate as, being long extinct and with no obvious “human-like” features or living descendants, there should be no intellectual bond between them and the pupils (e.g., they cannot have one as a pet). In all cases, we adopt or adapt lessons previously suggested in the literature.

While UK primary school evolution teaching presents a rare opportunity, there are at least four immediate caveats: whether primary school students are ready to understand evolution, whether we can test enough students for results to be robust, whether the results are replicable and whether, if teacher confidence is an issue, we can provide an implementation that reduces this as a covariate. Regarding the last issue, teacher confidence, we both consider this as a variable and provide in-school training to recruits, as part of our measure to maximise recruitment.

Regarding the first, one might reasonably question whether our target age group of 10–11-year-old students will be receptive to a concept as abstract as evolution. There is some *prima facie* evidence to suggest that children as young as five can grasp some ideas about genetics and natural selection if the correct type of instruction and scaffolding is provided^12^. More generally, some children are able to reason in evolutionary terms by the age of eight, reaching a transition phase between eight and nine when they start to understand microevolution in terms of intraspecific variation and confronting existential questions. Around age 10 some can understand (and accept) the continuity of species, this being the beginning of a macroevolutionary understanding^13^.

The second issue, testing a large enough number of students, is more problematic. To enable large sample sizes, we need an assessment methodology that is scalable. Indeed, while there are diverse activities, and a recognition of the need to identify the most effective evolutionary educational strategies and activities, there is very little quantitative evidence as to what works in the classroom^14^. This is in no small part due to a lack of assessment tools, which is particularly acute for primary-aged children where literacy issues can be problematic. The dearth of quantitative evidence obtained within a primary classroom setting is striking, there being only one existing study conducted with primary age students in the UK^15^. Consequently, the bulk of the existing research into school-age understanding of genetics and evolution focuses on secondary school children, with very little known about the understanding of evolution in primary school children. For a summary of existing studies see Supplementary Table 1.

Given this recognised lack of tools for the assessment of the understanding of evolution, particularly for use in primary students, we developed and benchmarked an easy to deliver quantitative assessment tool that could provide a scalable platform for large scale assessment^16^. Given the scalability of our tool, the third issue, replicability, is solved through dividing students into a test sample and a replicate sample. The test group (Tranche 1) has *N* = 1152 students from 17 schools, the replicate (Tranche 2) has *N* = 1505 students from 28 schools, although not all have testing both before and after the intervention.

While the recent introduction of evolution as a subject in UK primary schools provides an exceptional opportunity to consider the broader scale issues of teaching methodology, knowing how best to teach evolution specifically in this age group is important. There is growing recognition that young children benefit from studying evolution when biology is first introduced in primary school^8^ when they are most receptive to new ideas and are actively questioning how the world works. Evolution education in primary schools helps to establish the foundation for student understanding and to develop a deeper understanding during progression through future stages of the spiral National Curriculum^17^ with each subsequent exposure tending to increase the understanding of evolution.

We designed a large scale replicated randomised control trial (RCT) that employs our quantitative tool, supported by qualitative analysis. Our RCT is designed to ask (a) can students learn about evolution at this age group with effect sizes of improvements above implementation thresholds, (b) what modes of teaching approaches work best and (c) whether there are interactions between different teaching modes taught in series. We are unaware of RCTs looking for interaction effects between the modes of teaching that we explore.

While teaching the historical development of current evolution concepts has been shown to be beneficial and is congruent with cognitive development theory^18^, we do not adopt this structure. Rather, following evidence that teaching genetics/inheritance prior to teaching evolution (“Genetics-first”) improves understanding of both subjects^19^, we designed four schemes of work each consisting of four lessons taught in the same order: phenotypic variation/inheritance, natural selection, geological time and homology/common ancestry. Congruent with the age and ability of our novice students we employed a “phenotypes first” approach to introduce each scheme and act as the foundation for future improvements in conceptual understanding of genetics within a spiral curriculum. Lessons two (natural selection) and four (homology) were variable in their approach (as described above), leading to a 2 × 2 structure, each class being randomly assigned one of the orders. For specification of the four schemes see Table 1. For details of implementation see “Methods”. Given conventional wisdom we can also predict the expected success of each scheme of work (Table 2). Note that the 2 × 2 structure is the recognized highest power experimental design for the examination of interaction effects in drug trials (e.g. CHARISMA). We adopt drug trial recognised designs and CONSORT standards.

**Table 1:** Schematic outlining the content of the work phase activities for the four different SoW.

SoW	Lesson 1	Lesson 2	Lesson 3	Lesson 4
1	Quantitative investigation of variation of traits within the class.	Investigating natural selection in peppered moths. *Student-centred “hands on” hunting activity*	Investigating geological time. *Toilet roll timeline*	Investigating homology and common ancestry. *Non-human Trilobite activity*
2	Quantitative investigation of variation of traits within the class.	Investigating natural selection in peppered moths. *Student-centred “hands on” hunting activity*	Investigating geological time. *Toilet roll timeline*	Investigating homology and common ancestry. *Human-centred pentadactyl limb activity*
3	Quantitative investigation of variation of traits within the class.	Investigating natural selection in peppered moths. *Teacher-centred PowerPoint*	Investigating geological time. *Toilet roll timeline*	Investigating homology and common ancestry. *Non-human Trilobite activity*
4	Quantitative investigation of variation of traits within the class.	Investigating natural selection in peppered moths. *Teacher-centred PowerPoint*	Investigating geological time. *Toilet roll timeline*	Investigating homology and common ancestry. *Human-centred pentadactyl limb activity*

Lesson 1 and Lesson 3 were constant in all SoW. There were two different main activities in Lesson 2 and 4, giving a total of 4 different pathways for use in schools. In italics are the classification of the activity.

**Table 2:** Summary of the different main activities within the four SoW and their predicted effectiveness from existing research literature.

Scheme of Work	Main activity carried out by students		Predicted order of effectiveness
	Lesson 2	Lesson 4	
1	**Hunting paper moths**	Trilobites	=2^nd^
2	**Hunting paper moths**	**Pentadactyl limb**	1^st^
3	Power point and scaffolded written exercise	Trilobites	4^th^
4	Power point and scaffolded written exercise	**Pentadactyl limb**	=2^nd^

Highlighted activities (in bold) should be more effective given conventional wisdom. SoW 2 should be the most effective as it contains two activities predicted to be more effective. SoW 1 and 4 should be equally effective as both contain 1 activity predicted to be more effective. SoW 3 should be the least effective as it does not contain any of the predicted effective activities.

## Results

### The student assessment instrument was valid and reliable

We replicated our previous study^16^ demonstrating that our assessment tool is fit for purpose. Several different metrics were considered: internal reliability (Cronbach’s alpha), the correlation between paired pre and post-test scores, question fatigue, readability and the ability to discriminate between individual students. The results show that the assessment instrument was accessible, consistent, discriminatory and of appropriate difficulty for students of all abilities. They also showed no significant question fatigue with good reliability between data sets further validating our assessment instrument. See Supplementary Note 1 and Supplementary Figs. 1 and 2 for full details.

### Teaching interventions significantly improved student understanding (effectiveness of teaching H_1_)

**Fig. 1:**
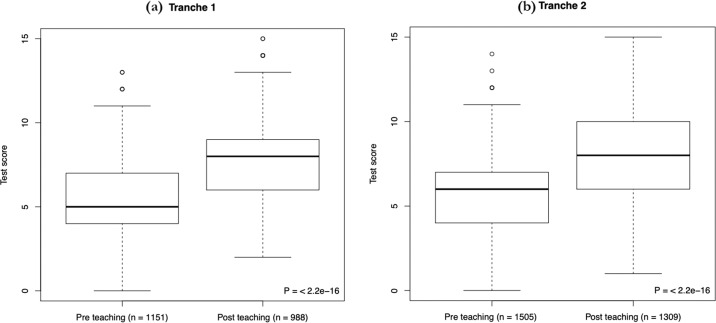
Box plots showing the difference between unpaired pre and post-tests for all students who participated in the study in both tranches. **a** Tranche 1: pre-test mean = 5.48 ± 2.13, post-test mean = 7.92 ± 2.38; **b** tranche 2: pre-test mean = 5.72 ± 2.27, post-test mean = 8.09 ± 2.70.

**Fig. 2:**
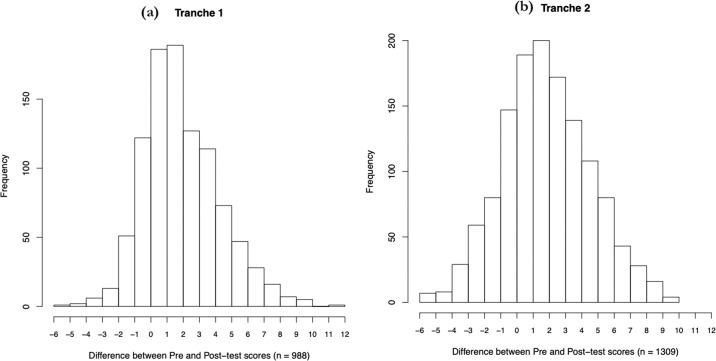
Histograms showing the distribution of the change in scores for students taking both pre and post tests for both tranches. **a** Tranche 1: pre-test mean = 5.51 ± 2.15, post-test mean = 7.92 ± 2.38; **b** tranche 2: pre-test mean = 5.77 ± 2.25, post-test mean = 8.09 ± 2.70.

Do the Schemes of Work (SoW) considered *en masse* improve student understanding? Using Wilcoxon rank-sum tests we addressed this issue in two modes. First, we compared all pre-test and post-test scores in an unpaired manner, enabling us to employ all data (Fig. 1). The mean student test score increased significantly in tranche 1 by 2.44 marks (16.27%, *P* < 2.2 × 10^−16^) with a large effect size (Cliff’s *d* = 0.55, 95% CI: 0.52–0.59). This was replicated in Tranche 2 (increase in mean by 2.37 marks, 15.80%, *P* < 2.2 × 10^−16^, Cliff’s *d* = 0.49, 95% CI: 0.45–0.52). Second, to control for the performance of any given student, we considered the distribution of the change in score values for all students who took both the pre and post-test assessments (Fig. 2). The mean student change in score was +2.42 marks (+16.13%, *P* = < 2.2 × 10^−16^, Cliff’s *d* = 0.55, 95% CI: 0.51–0.59) in tranche 1, replicated in tranche 2 (mean increase 2.32 marks, 15.47%, *P* = < 2.2 × 10^−16^, Cliff’s *d* = 0.48, 95% CI: 0.44–0.52).

Effect size is a useful way to assess the effectiveness of a particular intervention as it enables the measurement of both the *improvement* (gain) in learner achievement within a group of learners and the *variation* of student performances expressed on a standardised scale. From meta-analysis of educational interventions, Hattie^20^ determined a ‘hinge point’ effect size of 0.40 could be used as a guide to the effectiveness of educational interventions, with above 0.40 being effective but lower than 0.40 needing further consideration or modification. We note that not only is there improvement in both tranches, but the effect sizes, determined by Cliff’s d, are well above threshold for implementation (tranche 1 = 0.55, tranche 2 = 0.48). Additionally, using the more limited results obtained from students who took all three tests (tranche 1: *N* = 320; tranche 2: *N* = 523) we showed that the teaching interventions had some degree of long-term retention, although some of this understanding waned over time (see Supplementary Note 2 and Supplementary Fig. 3 for more detail).

### All SoW produced significant improvements in student understanding (effectiveness of individual SoW H_1_)

**Fig. 3:**
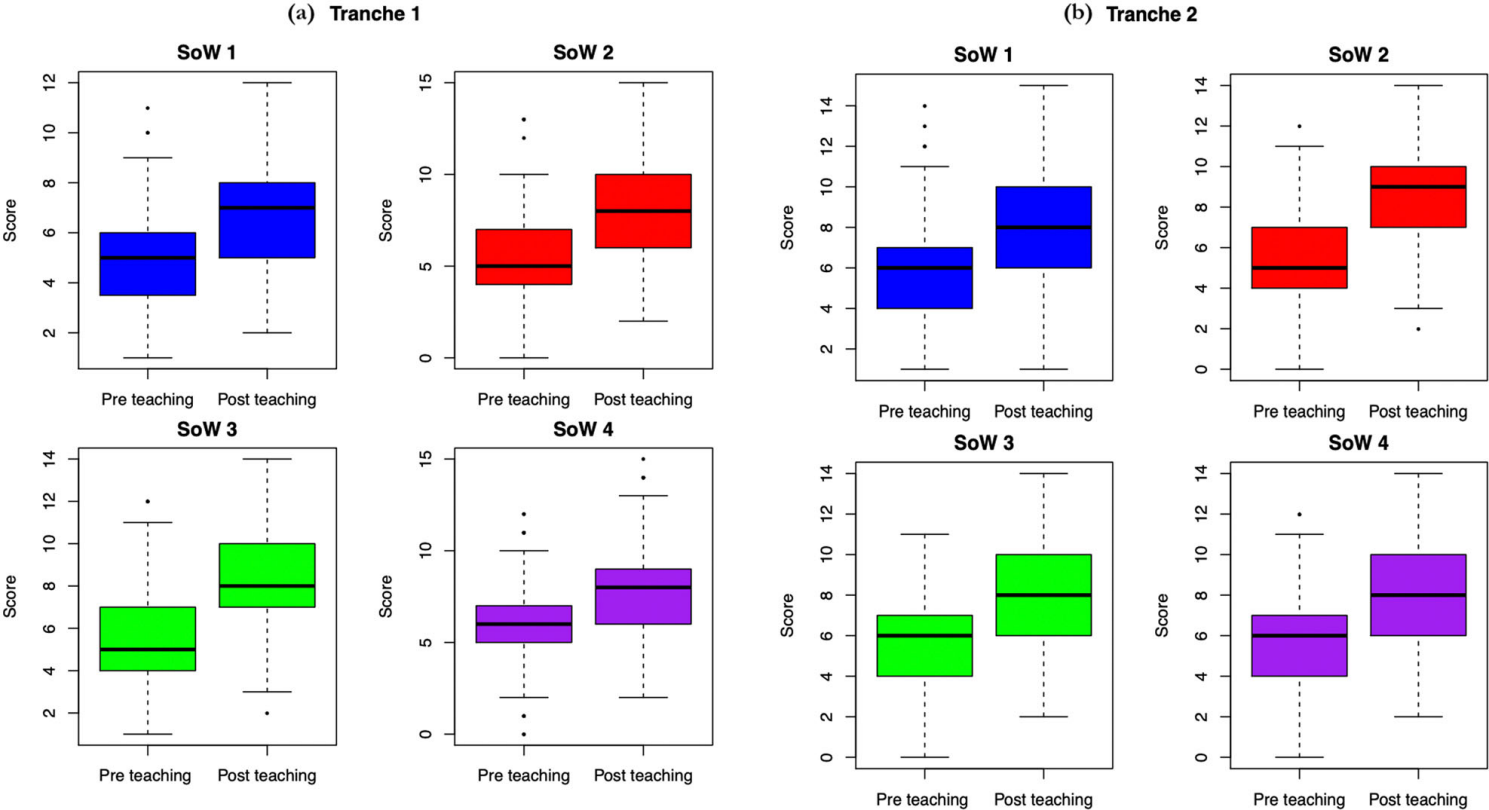
Box plots showing the difference between matched pre and post-tests in both tranches of data stratified by SoW. **a** Tranche 1; **b** tranche 2.

Does each SoW considered in isolation improve understanding? Comparison of the paired post and pre-test scores for individual students, showed significant improvement for each of the 4 SoW in both tranches (*P* < 2.2 × 10^−16^, Wilcoxon signed-rank test in each case, Fig. 3). Additionally, each SoW improved performance with moderate to large effect sizes (Table 3), values being above the 0.4 effectiveness threshold, except for SoW1 in Tranche 2 (Cliff’s *d* = 0.38).

**Table 3:** Values of Cliff’s d (effect size) for the difference between paired pre and post test scores for each SoW in both tranches of data.

SoW	Tranche 1			Tranche 2		
	Cliff’s *d*	95% CI	Size	Cliff’s d	95% CI	Size
1	0.44	0.33–0.54	Moderate	0.38	0.31–0.45	Moderate
2	0.55	0.47–0.62	Large	0.66	0.58–0.72	Large
3	0.66	0.58–0.72	Large	0.51	0.43–0.59	Large
4	0.52	0.44–0.60	Large	0.42	0.34–0.49	Moderate

Note all *P*-values <2.2 × 10^−16^ for the individual Wilcoxon signed-rank tests.

Before any further analysis to determine predictors of student responses (involving the difference between pre and post-test scores), the data were adjusted by correcting for pre-test scores, the uneven distribution of raw test scores between the four SoW and to mitigate the “ceiling effect”, a common quantitative assessment problem. Unlike measures of normalised gain, which make assumptions about the relationship between the change in score and the pre-test score, all subsequent analyses in this study employ LOESS residual scores that derive the relationship from a non-parametric regression (see Supplementary Note 3 for more detail).

### Results of tests between alternative teaching modes are weak and un-replicated

Our 2 × 2 structure enables us in the first instance to ask, when considering the alternatives for Lessons 2 and 4 in isolation, whether one teaching mode is better than the other. Educational research predicts that in Lesson 2 the student-centred “hands-on” hunting moths activity would be more successful than the scaffolded teacher-centred PowerPoint activity. Similarly, in Lesson 4 the pentadactyl limb activity is expected to be more effective.

To evaluate teacher-centred against student-centred activity, we contrast the performance through Lesson 2 by comparing schemes 1 and 2 (student-centred moth hunting) with schemes 3 and 4 (PowerPoint moth lesson). Results are inconsistent. In tranche 1, the lesson expected to be more effective (student-centred pecking moths), was the least effective, albeit with small effect size (Table 4). Conversely, in tranche 2 the result was reversed but the effect size is weaker still and not significant. The significance in the first trial survives multiple testing (*P* = 0.05/2). These results do not support educational research that “hands on” activity aids understanding.

**Table 4:** Results of Wilcoxon rank-sum tests comparing the difference between LOESS residual scores of student-centred and teacher-centred activities in Lesson 2 with Cliff’s d effect size values.

Tranche	*P*-value	Cliff’s *d*	Mean ± SD SoW 1 and 2 (student-centred)	Mean ± SD SoW 3 and 4 (teacher-centred)
1	1.80 × 10^−5^	−0.21 95% Cl: −0.30 to −0.11 (small)	−0.69 ± 2.74 *N* = 485	0.46 ± 2.96 *N* = 503
2	0.15	0.06 95% Cl: −0.02 to 0.16 (negligible)	0.32 ± 3.40 *N* = 710	8 × 10^−3^ ± 3.55 *N* = 599

To evaluate human-centred against the non-human centred activity (Lesson 4), we compare the performance of schemes 1 and 3 (trilobites) with 2 and 4 (human-centred pentadactyl limb) (Table 5). Results are in the expected direction in both tranches but in both the effect size is “negligible” (not significant in tranche 1 and the weak significance in the replication (*P* = 0.04) is sensitive to multi-test correction (*P* > 0.05/2).

**Table 5:** Results of Wilcoxon rank-sum tests comparing the difference between LOESS residual scores of non-human and human-centred activities in Lesson 4 with Cliff’s *d* values (effect size).

Tranche	*P*-value	Cliff’s *d*	Mean ± SD SoW 1 and 3 (non-human)	Mean ± SD SoW 2 and 4 (human)
1	0.10	0.08 95% Cl: −0.18 to 0.01 (negligible)	−0.37 ± 2.73 *N* = 412	0.023 ± 3.067 *N* = 576
2	0.04	0.09 95% Cl: −0.17 to −3 × 10^−3^ (negligible)	−0.13 ± 3.63 *N* = 700	0.350 ± 3.115 *N* = 609

### The SoW predicted to be the least effective was highly effective (effectiveness of individual SoW H_1.0_)

**Fig. 4:**
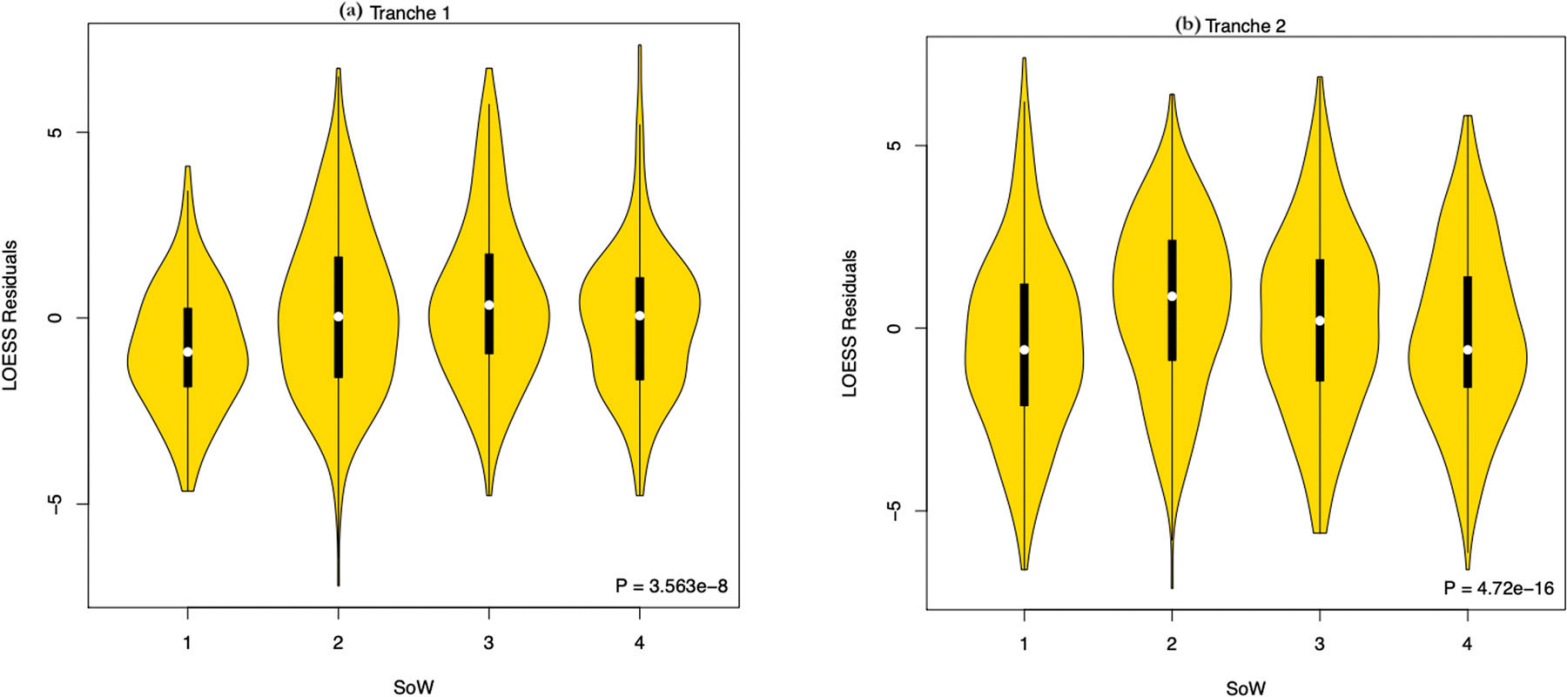
Violin plots showing LOESS residuals stratified by SoW for both tranches. **a** Tranche 1: SoW 1: mean = −0.83 ± 1.65, median = −0.91, SoW 2: mean = 0.16 ± 2.25, median = 0.04, SoW 3: mean = 0.47 ± 2.19, median = 0.35, SoW 4: mean = −0.07 ± 1.93, median = 0.06; **b** tranche 2: SoW 1: mean = −0.40 ± 2.51, median = −0.60, SoW 2: mean = 0.67 ± 2.34, median = 0.85, SoW 3: mean = 0.198 ± 2.48, median = 0.21, SoW 4: mean = −0.191 ± 2.40, median = −0.60.

We next considered the optimal teaching scheme (Table 2), predicting the most optimal to be student-centred for Lesson 2 and human-centred for Lesson 4 (SoW 2) whereas the least optimal should be teacher-centred and non-human (SoW 3). We thus predict heterogeneity between the SoWs and a rank order of 2 < 1 = 4 < 3 (Table 2). We consider the relative performance of all four schemes with the added advantage that it can expose any hidden interaction effects. Indeed, the above analyses assume that there is no interaction between modes of teaching in Lessons 2 and 4. There is significant heterogeneity in the relative effectiveness of the four SoW, seen in both tranches of data (tranche 1: *χ*^2^ = 37.53, *P* = 3.56 × 10^−8^; tranche 2: *χ*^2^ = 40.91, *P* = 6.84 × 10^−9^, Kruskal–Wallis rank-sum test on residuals with Dunn post-hoc and Bonferroni correction) (Table 6; Fig. 4). We conclude that the four SoW do not have the same effectiveness.

**Table 6:** Summary of Bonferroni adjusted *P*-values obtained from the Kruskal–Wallis rank-sum test with a Dunn post-hoc of LOESS residual scores from the four SoW in both tranches.

Tranche 1	SoW	1 (*N* = 171)	2	3
	2 (*N* = 314)	**6.72** **×** **10**^**−6**^ **[1** **<** **2]**	–	–
	3 (*N* = 241)	**3.29** **×** **10**^**−9**^ **[1** **<** **3]**	0.80 [2 < 3]	–
	4 (*N* = 262)	**8.31** **×** **10**^**−4**^ **[1** **<** **4]**	1.00 [4 < 2]	0.09 [4 < 3]
Tranche 2	SoW	1 (*N* = 422)	2	3
	2 (*N* = 288)	**8.53** **×** **10**^**−9**^ **[1** **<** **2]**	–	–
	3 (*N* = 278)	**1.88** **×** **10**^**−3**^ **[1** **<** **3]**	0.07 [3 < 2]	–
	4 (*N* = 321)	1.00 [1 < 4]	**1.84** **×** **10**^**−5**^ **[4** **<** **2]**	0.27 [4 < 3]

Note significant results are highlighted in bold.

A subsidiary question is whether the relative effectiveness of any given SoW is in itself replicable. We expect the order to be 2 < 1 = 4 < 3. In tranche 1, the order of relative effectiveness of the schemes is 1 < 4 < 2 < 3 and in tranche 2, the order was 1 < 4 < 3 < 2 (Table 6). The most striking insight here is that the scheme we expected to be the least effective (SoW 3) was either the most effective or the next most effective. Strikingly, in neither tranche do we find a significant difference between SoW 2 and 3 (Table 6), the two we expected to be most and least effective, respectively.

The other curious result is that in both tranches, SoW 1 (student-centred, non-human focus) is not simply the least effective but is significantly less effective than other schemes (except for the 1–4 comparison in tranche 2). However, it should be noted that even though Scheme of Work 1 was the least effective relative to the other schemes it significantly improved student performance in both tranches (*P* < 2.2 × 10^−16^, Wilcoxon signed-rank test) with moderate effect sizes (see Table 3).

### Interaction effects are needed to explain the impacts of the schemes (interaction effects H_1_)

At first sight the above results present an enigma. When comparing methods within Lesson 2 or within Lesson 4 we see marginal or non-replicable results. By contrast, analysing the same data as four separate SoW, we see repeatable and significant differences. This indicates potentially important interaction effects (IEs), wherein combinations of pairs of lessons achieve more or less than when considering in an unpaired manner.

Using the method of Sevdalis and Jacklin^21^, we computed the interaction effect sizes for each of the Schemes of Work. A positive IE indicates that the combination of two lessons has a positive effect on overall score outcome. We first ask whether, in general, the lesson combinations had a significant influence on post-teaching scores than expected simply by chance. To do this, we calculated the sum of absolute interaction effects (AIS) for each SoW. We then compared the true AIS against the simulated AIS for 10,000 random simulants generated using the same underlying data, but where the SoW for each participant was randomly assigned (Table 7). In both tranches, the true AIS is significantly greater than that expected by chance (*P* ~ 1.00 × 10^−4^, one-tailed empirical *P*-value) with no simulant exceeding the observed interaction. This suggests the unique combinations of lessons in each SoW have meaningful impacts on scores.

**Table 7:** Comparisons of true absolute interaction effect sums with the equivalent values for data in which the Schemes of Work have been randomly assigned.

Tranche	True AIS	Minimum simulant AIS	Median simulant AIS	Maximum simulant AIS	One-tailed Empirical P
1	1.525	1.34 × 10^−5^	0.185	1.122	9.99 × 10^−5^
2	1.452	2.88 × 10^−6^	0.185	1.244	9.99 × 10^−5^

We can also quantify the differences between the real IE and simulant IEs for the four SoW separately. In both tranches and in all SoW, the true interaction effect was significantly different to the simulant IEs (Table 8). Notably, for SoW 2 and 3, our two most successful schemes, the direction of the interaction term is positive, implying both outperform expectations drawn from analysis of the underlying alternatives when these are considered in isolation. We conclude that consideration of teaching in series exposed a highly repeatable interaction between modes of teaching.

**Table 8:** Comparison of true interaction effects with the equivalent values for data in which the Schemes of Work have been randomly assigned.

Tranche	SoW	True IE	Minimum simulant IE	Median simulant IE	Maximum simulant IE	One-tailed Empirical P
1	1	−0.381	−0.252	−6.798 × 10^−4^	0.280	9.99 × 10^−5^
	2	0.381	−0.280	6.798 × 10^−4^	0.252	9.99 × 10^−5^
	3	0.381	−0.280	6.798 × 10^−4^	0.252	9.99 × 10^−5^
	4	−0.381	−0.252	−6.798 × 10^−4^	0.280	9.99 × 10^−5^
2	1	−0.363	−0.271	7.558 × 10^−4^	0.311	9.99 × 10^−5^
	2	0.363	-0.311	−7.558 × 10^−4^	0.271	9.99 × 10^−5^
	3	0.363	-0.311	−7.558 × 10^−4^	0.271	9.99 × 10^−5^
	4	−0.363	-0.271	7.558 × 10^−4^	0.311	9.99 × 10^−5^

### Ability, but not age or gender, repeatedly predicts gain in understanding

While above, we have shown both interaction effects and SoW effects, there is variation that remains unexplained. What could explain the variation in the students’ response to teaching and, might any such covariates explain the unexpected success of SoW 3? Using a multivariate model exploring the combined effects of gender, age, ability and SoW we can account for 7.10–8.30% of the total variance in student score across both tranches of data. Results indicate that age was not a significant variable but students with higher science ability (as judged by their teachers) improved more. The results also showed weak and non-replicable gender effects which supports the conclusion of the National Pupil Database^22^. See Supplementary Note 4 for more details. The multivariate analysis also confirms that SoW 1 was the least effective relative to the other schemes in both tranches. These results suggest that Schemes of Work 2 and 3 are the most effective teaching intervention programmes, even after allowing for covariates.

### Class-level confirmation of student order of alternative teaching mode effectiveness (effectiveness of individual SoW H_1_, interaction effects H_1_)

To measure the contribution of teacher effectiveness in the classroom, class-level analyses were carried out using mean class LOESS residual score, then matched to completed teacher questionnaires (tranche 1: *N* = 27; tranche 2: *N* = 43). Completion of the teacher questionnaires was voluntary with 89.74% teachers participating over the two tranches. Classes without a corresponding completed teacher questionnaire were omitted from these analyses.

Class-level analysis of the effectiveness of the four schemes mirrored the pattern of SoW effectiveness found at the student level (Table 9) with moderate effect sizes for these differences (*ɛ*^2^ = 0.14, tranche 1 and *ɛ*^2^ = 0.13, tranche 2). However, we found no significant differences between SoW and LOESS residual scores at teacher level in either tranche, likely to be caused by the small sample size.

**Table 9:** Table summarising teacher level LOESS residual scores stratified for SoW.

Tranche	SoW	Mean and SD	Relative effectiveness
1	1 (*N* = 7)	−0.71 ± 0.23	4th
	2 (*N* = 13)	0.11 ± 1.01	2nd
	3 (*N* = 10)	0.33 ± 1.12	1st
	4 (*N* = 10)	−0.13 ± 0.83	3rd
2	1 (*N* = 17)	−0.47 ± 1.31	4th
	2 (*N* = 13)	0.46 ± 0.94	1st
	3 (*N* = 12)	0.30 ± 1.45	2nd
	4 (*N* = 14)	−0.27 ± 0.97	3rd

### Poor replicability of teacher-level predictors of student performance

Given the correlation between acceptance and understanding, might teacher attitudes to evolution influence their teaching and student performance? To evaluate repeatability, or amassed best evidence more generally, of teacher-level predictors of student performance, we take several approaches: combined P-values with multi-test correction, replication of significance and correlation of effect sizes.

First, as an assessment of amassed best evidence, we consider the combined *P*-value from a total of 11 predictor factors by considering Fisher’s method of *P* value combination, with *P* values drawn from the two independent tranches (Supplementary Table 2). We identify three factors as being significant: a teacher’s years of experience, their perceived improvement in confidence to teach the topic and whether or not they completed their pre-teaching questionnaire (completion bias). All three factors are possibly related to teacher confidence either directly or indirectly. However, after Holm multi-test correction (with 11 predictors) these predictors are insignificant, save the increased confidence.

A second mode of analysis isolates any results that were significant in the first tranche and asks whether they are also significant in the second, with multi-test correction dependent on the number of variables passing into the replication test. Only one result that was significant in tranche 1 was also significant in tranche 2 (increase in confidence; *P* = 3.40 × 10^−2^ and *P* = 2.00 × 10^−3^, respectively). We conclude that for the most part results cannot be replicated, but training to improve teacher confidence can be easily defended.

**Fig. 5:**
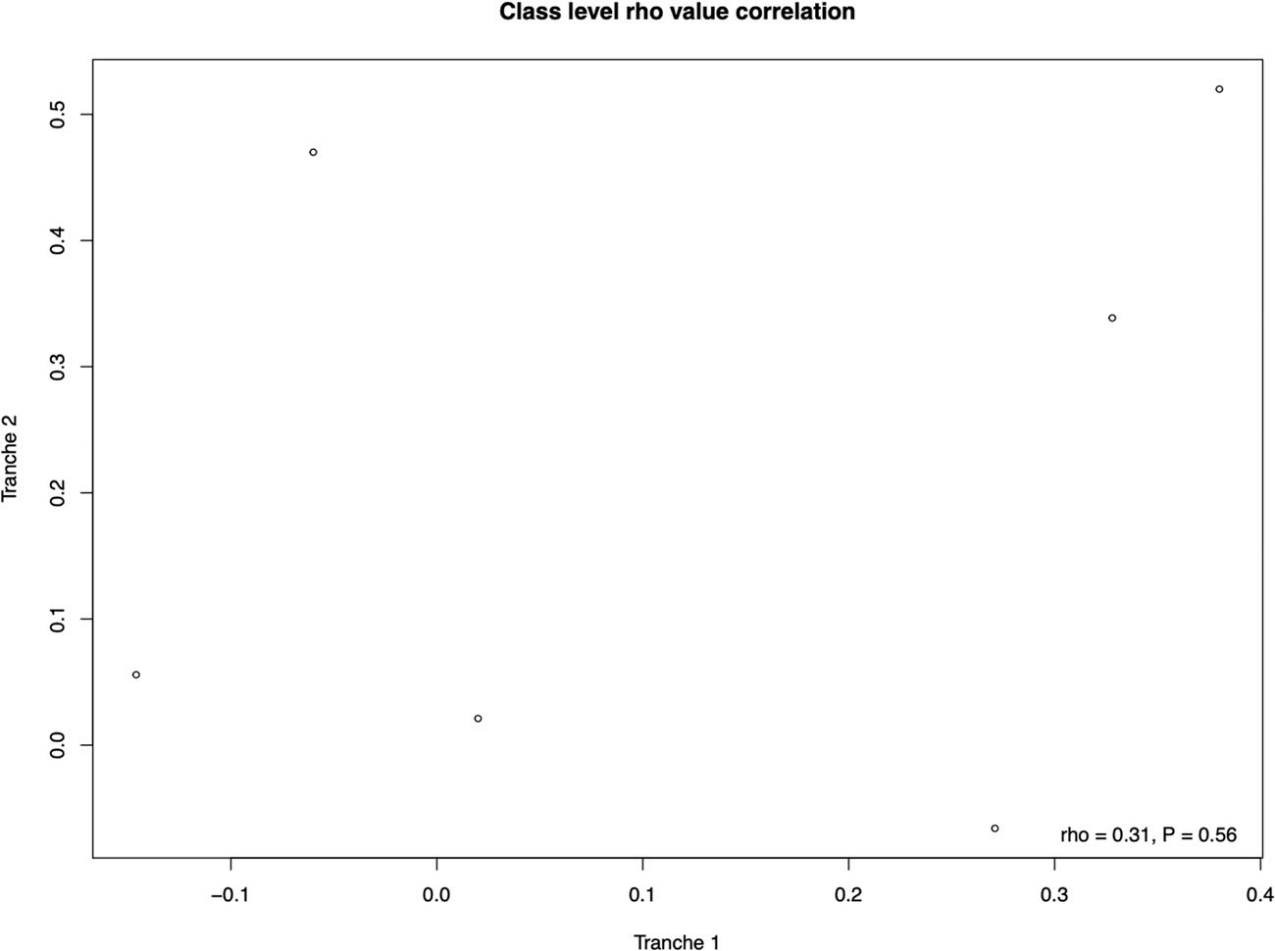
Scatterplot showing the correlation between rho values obtained from class level analyses between both tranches.

The above approaches are focused at uncovering significant predictors of improvement in scores. We can ask a more general question, namely whether our class-level factor results show reproducibility between data sets. If so, even if non-significant, we would expect that values of *P* and rho taken from the individual equivalent statistical tests would be positively correlated. We find no significant correlation between *P* values (*ρ* = 0.41, *P* = 0.19, Spearman’s rank correlation coefficient, *N* = 11) nor between rho values (*ρ* = 0.31, *P* = 0.56, Spearman’s rank correlation coefficient, *N* = 6) obtained in the individual class level tests carried out to identify possible predictors of performance (Fig. 5), although both correlations had moderate effect size. We conclude that there is no significant replicability of result effect sizes.

### SoW 3 is the most effective in the replicate after controlling for biased distribution of teacher characteristics. (effectiveness of individual SoW (H_1,_ H_1.0_), interaction effects (H_1_)

Above we identified only one teacher-level predictor that is replicable. Can the differences between the four SoW be explained by a biased distribution of teacher characteristics rather than a property of the schemes themselves? Perhaps after any such correction, SoW 3 will be found to be successful simply because the teachers were especially effective.

First, we ask whether any teacher characteristic is different between the schemes. Employing Kruskal–Wallis rank-sum tests we find no significant difference in the distributions of years of experience (tranche 1: *P* = 0.23, tranche 2: *P* = 0.22), completion bias (tranche 1: *P* = 0.07, tranche 2: *P* = 0.18), understanding (tranche 1: P = 0.59, tranche 2: *P* = 0.56) or acceptance of evolution (tranche 1: *P* = 0.52, tranche 2: *P* = 0.14) between the four SoW.

**Fig. 6:**
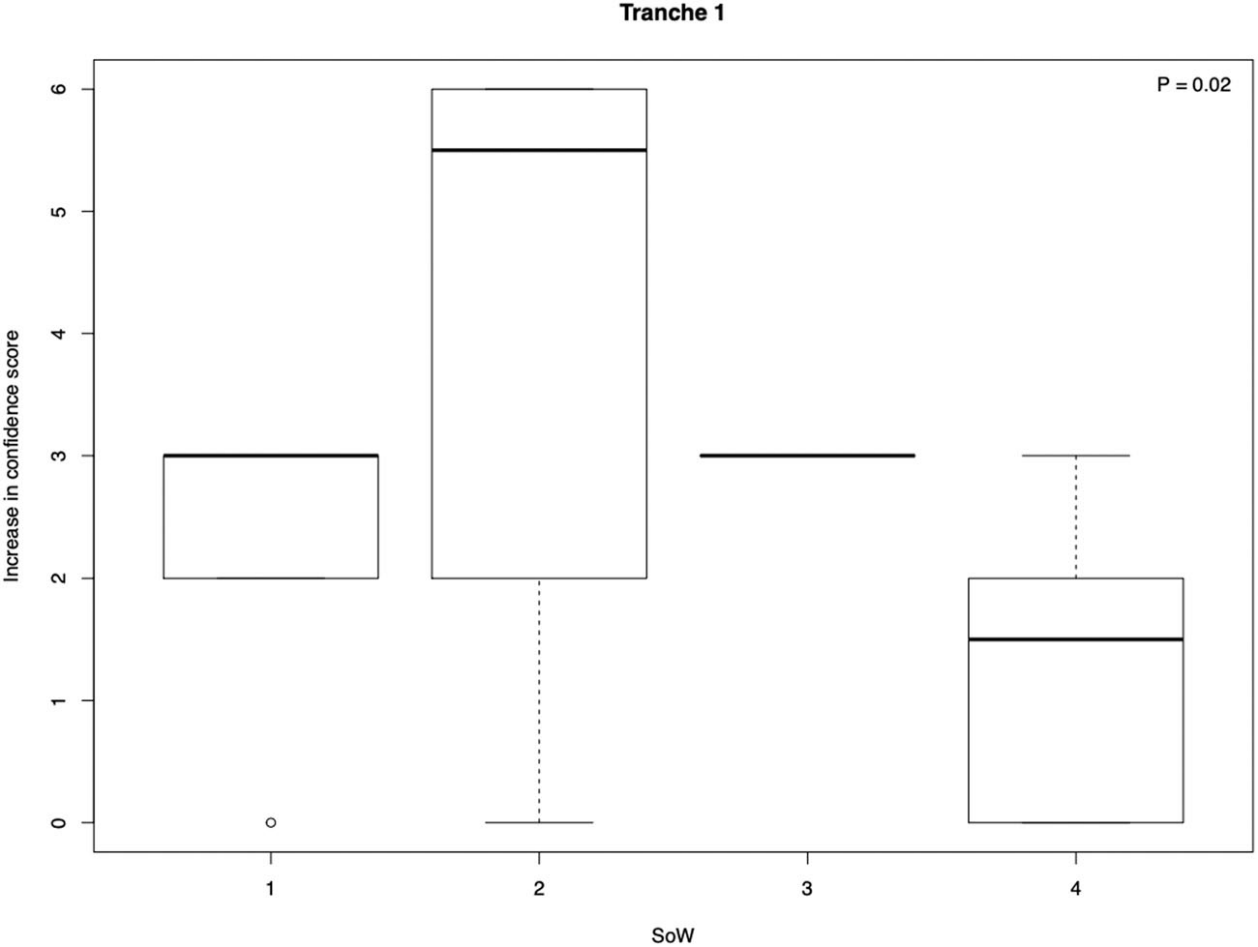
Boxplots showing the relationship between the distribution of teacher increase in confidence scores for teaching evolution stratified for SoW in tranche 1. Note; Kruskal–Wallis test.

We find the distribution of perceived increase in confidence level was significantly different for the SoW in tranche 2 (Fig. 6) with a moderate effect size (*ɛ*^2^ = 0.24), but not for tranche 1 (tranche 1: *P* = 0.44, tranche 2: *P* = 0.02). This is a concern as this was also the replicable predictor. To address this concern, we consider a multivariate analysis in which the four schemes are considered as factors and students are assigned a score that is their teacher’s increase in confidence. We consider gender, age and ability as before. In tranche 1, as expected given that teacher confidence was not biased, results are unchanged (girls do better than boys, *P* = 0.001; higher ability students do better, *P* = 2.3 × 10^−6^; schemes are in the order 3 > 2 > 4 > 1). Importantly, on allowing for teacher improved confidence, the rank order of schemes in the replicate test now switches to 3 > 2 > 1 > 4. In the replicate, only student ability is a significant predictor (*P* < 1.7 × 10^−11^). Thus, allowing for a bias in the allocation of teachers in the replicate tranche supports that conclusion that the scheme expected to be least effective (SoW 3) is the most effective.

### Acceptance and understanding of evolution amongst primary school teacher cohort is high but only weakly correlate

To contextualise our results, it should also be noted that teachers in both tranches demonstrated high acceptance of evolution and a positive correlation between teacher acceptance of evolutionary theory and the understanding of natural selection. However, as observed in our prior analysis of secondary school students^19^, this correlation is notably weak (see Supplementary Note 5 and Supplementary Fig. 4).

### There are no significant repeatable school-level predictors

School-level analyses were carried out to compare the performance of individual participating schools and check for any unintentional school biases (tranche 1: *N* = 17, tranche 2: *N* = 28). Mean school LOESS residual scores were correlated with data gathered from the school’s most recent Ofsted publication, as well as data taken from the English Indices of Deprivation. Values of the indices were obtained using the school’s post code and it was assumed students attending a school lived within (a) the catchment area and (b) the same LSOA (Lower layer Super Output Area, or neighbourhood).

As above, we consider three modes of assessment of replicability. Under the replication of significance mode, we had no results significant in the first trial and so nothing to replicate (Supplementary Table 3). Under the combined *P*-values mode, there is only one significant result, namely primary schools out-perform middle schools (combined *P* = 0.03, Fisher’s test) with a large effect size in tranche 2 (Cliff’s *d* = 0.73). This result is sensitive to multi-test correction. Reanalysis of multivariate analysis excluding either middle schools or primary schools doesn’t affect prior results (Supplementary Note 6).

**Fig. 7:**
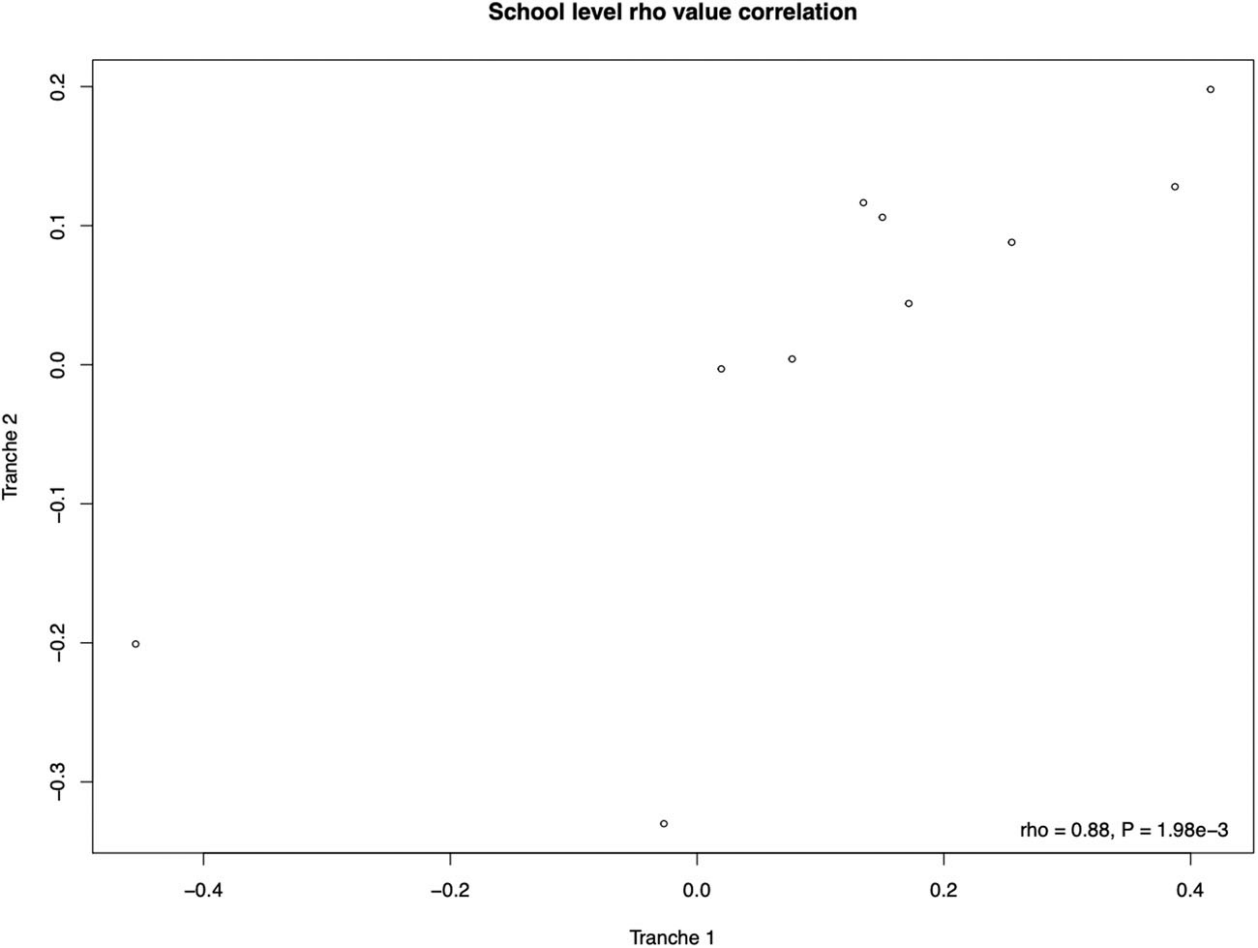
Scatterplot showing the correlation between rho values obtained from school level analyses in both tranches.

Although we find low replicability of significant predictors at school-level, the third mode of analysis, replicability of effect sizes, showed strong repeatability. We found a significant correlation between rho values (*ρ* = 0.89, *P* = 1.98 × 10^−3^, Spearman’s rank correlation coefficient, *N* = 10) obtained in the individual school-level tests carried out to identify possible predictors of performance (see Fig. 7). Both correlations had large effect sizes, the consistency of these results showing evidence of repeatability at this level of analysis. We could identify no robust significant school-level predictor, but could identify robust replicability of effect sizes.

### Teacher endorsement of all SoW

While SoW 2 and 3 appear to be particularly successful, their implementation requires their application to be amenable to teachers. Post-teaching teacher feedback (Supplementary Note 7) reveals that all four variable individual main activities for lessons 2 and 4 were successful in engaging the students in positive learning experiences. The choice of organisms was also appropriate to illustrate the complex concepts being introduced (Supplementary Note 8).

## Discussion

The “ecological niche” provided by the introduction of the Inheritance and Evolution topic into the UK Key Stage 2 National Curriculum in 2014 provided this study a unique opportunity to research teaching modes. We identified replicable heterogeneity between teaching modes but in a manner not obviously consistent with educational theory. The deviation from expectations in some part reflects an interaction effect, visible through our 2 × 2 serial testing. These results both question the consensus on the optimal teaching mode and, as with drug interaction studies, suggest that parallel testing regimes can miss quantitatively important effects. More prosaically, all the schemes were successful with above implementation threshold effect sizes. Importantly, students of all abilities (as judged by their teachers) responded positively to the teaching interventions, with evidence for longer term retention.

In finding that the students on average responded well to the teaching (with above threshold effect sizes in both replicates), our data supports the growing body of research suggesting that children aged 9–11 possess the cognitive ability to successfully understand the concepts of natural selection and evolution when provided with appropriate resources and teaching instruction^23^. It also supports the premise that primary students can successfully learn about natural selection and understand the basics of the principle if appropriate scaffolding is provided^24^.

We designed our SoW not simply to test a set of hypotheses but also, if viable, to be implemented easily and at minimal costs. The activities can be used in any standard (i.e. non-lab) classroom in a practical and (hopefully) interesting way. That the resources are simple, cheap and employ widely available resources, with above threshold effect sizes, implies easy and effective implementation.

A large scale in-school experiment of this variety comes with intrinsic limitations in its generalizability, its implementation and thus conclusions that can be drawn. Regarding generalizability related to populations a significant concern is school sampling. While the socio-economic profile of the schools was heterogeneous, participants were predominantly white of European or British descent, based in the Southwest of England. Our conclusions therefore cannot be extended to other racial groups or to hyper-religious contexts, where the teachers’ view of evolution may be substantially different. As schools had to volunteer to participate in the study, the sample was probably non-randomly biased towards well motivated classroom practitioners. We also note a caveat in our 2 × 2 design. Unlike a classical drug interaction trial, the nature of this study excludes any temporal effects because the simultaneous delivery of the SoW was not feasible.

While teacher delivery, as opposed to researcher/academic delivery, is a strength of our methodology, rendering it more relevant to the real school environment, it has the disadvantage that some teachers may not have adhered fully to any SoW. A possible upside of any bias towards more motivated teachers might conversely be good adherence to the lesson plan. Indeed, we detected only low levels of non-compliance (e.g. 6.8% of teachers failed to complete the final task). Statistically, so long as such non-adherence is random with respect to SoW, this would factor as a noise variable in any analysis. To this end we find no evidence that non-compliance differed between SoW or tranches from the qualitative feedback obtained from the teachers. Given the replicability of the strong performance of SoW 2 and 3, and the underperformance of SoW 1, we have reason to suppose that non-adherence does not provide a viable hypothesis to explain the variation between schemes.

An important decision in any pre-post testing approach is whether to ask the same questions or similar questions in both. We opted to use the same question commonly applied to minimize the possibility of a “similar” question not being similar as intended (see “Methods”). Importantly, in analyses comparing the different SoW, the testing regime is a fixed variable and so cannot explain any heterogeneity nor the interaction effect (residuals from the LOESS regression provide a mean of zero as all change is made relative to the median for the pre-score level). By contrast, however, if there is a learnt component to the answers provided post testing that is conditional on the first round of testing, then we cannot strictly claim an absolute improvement amongst the students. However, with no source of reinforcement, and indeed the possibility of negative effects (see “Methods”), we see no reason to suppose that actual improvement isn’t the most parsimonious interpretation of the tendency for raw performance scores to improve. The qualitative data from both the students and teachers also support the notion that the students did indeed gain understanding in absolute terms.

In determining the predictors of gain in understanding, our metric of teacher assessment of student ability has the advantage of deriving from an individual best acquainted with the student, but may be subject to teacher bias and error^25^. Despite this, meta-analysis of the effect sizes from 75 studies over the last 20 years by Südkamp, Kaiser and Möller^26^, found a positive and strong correlation (*r* = 0.53) between teacher judgements of ability and actual student test performance, as expected by cognitive theory^27^. This is consistent with our correlation. It also supports the premise that teacher judgements of ability were both largely valid and reliable for use in this study.

Aside from the large scale RCT nature of the trial, we were aware of the “replication crisis” problem in science, known to be acute in psychology^28^, in which the results of studies cannot be reproduced on subsequent investigation. To this end we included a large-scale replication (tranche 2 using a sample size larger than the original tranche 1). Our replication data showing that all four SoW improve understanding, offer a robust conclusion. Additionally, it is striking that an interaction effect is also strongly replicated. In this context, we recommend SoW 2 and 3 could be well defended as effective methods of delivery. By contrast, very few of the results relating to student performance at the teacher/class level were statistically significant in either tranche despite the unusually large sample sizes. Given this lack of replicability in two unusually large populations, we caution against the derivation of policy from small effect size trends in un-replicated trials.

Unexpectedly the teacher-centred peppered moth PowerPoint activity aligned with studying homology in Trilobites was the most effective scheme. The success of the combined package is contrary to common educational discourse and is also unexpected given the poor replicability and weak effects of the alternatives considered in isolation. The latter enigma is resolved by recognition of a replicable interaction effect. Such an effect has a statistical definition but must also have an underlying cause. Whilst the cause of this interaction cannot be identified by our study, the design of this study allows a number of factors to be controlled and excluded. First, the nature of lesson allocation means a teacher’s choice of lessons to teach (and possible associated covariates) is not a factor. It is also unlikely to be caused by the uneven distribution of “better’ teachers across the schemes as control for the one important variable only reifies the success of SoW 3. As we used the peppered moth to show the process of natural selection in Lesson 2 in both alternative activities, the difference cannot be due to the context of the activity but rather the nature of the activity carried out (student or teacher-centred). Conversely, in Lesson 4 the same activity was employed to investigate common ancestry through homology but this time in two different contexts (extinct Trilobite species and extant mammalian pentadactyl limbs) and so the difference cannot be due to the nature of the activity but caused by the context of the activity.

Rather, the results are consistent with the possibility that the main activity in Lesson 2 acts as a primer for the main activity in Lesson 4. These results suggest that the pairs of main activities contained within the sequences of lessons of the two more effective SoW acted in a reciprocal and reinforcing manner within the sequences of lessons. Improvements in one type of knowledge thus support improvements in another type of knowledge, supporting further improvements in the first and so on. Further work is required is establish the commonality of such interaction effects in serial lessons in other contexts. More generally, our results underpin the notion that parallel modes of testing, even in an RCT format, might mislead and that lessons plans in series should be considered for fuller testing. We can only speculate as to why we observe interaction effects and why the unexpected effectiveness of SoW 3 was highly effective when it should have been the least effective. Perhaps the more logically ordered teacher-centred PowerPoint moth activity and worksheet interacted positively with existing student interest in fossils when they studied homology and common ancestry in Trilobites? The success of SoW 3 appears to contradict the current discourse around active, human-centred learning, suggesting a review of these dichotomous perspectives of learning policy is needed. A concept-based curriculum approach, which fosters a deeper understanding through an iterative, “thinking-centred” approach^29^ may integrate such binary perspectives and support pedagogic practice among teachers to cultivate a long-term conceptual understanding of evolution. With school and college curricula being restructured based on the current wisdom, our results suggest further scrutiny is called for to identify what actually works, for whom, and under what conditions.

While the cause of the interaction effect remains uncertain, that the individual components of SoW 3 might be successful does not itself contradict the notion that engagement is important for effective learning. From qualitative analysis (Supplementary Notes 7 and 8) we discerned, for example, that the trilobite activity engaged the students in no small part because it wasn’t human: teachers spoke of the students being engaged with the fossils and fascinated by them in part because of their strangeness (fascination with dinosaurs in this age group is similarly common). Using a teacher-centred story book approach also accords with the notion that an engaging narrative can be an effective communication tool (e.g. Kelemen, Emmons, Seston Schillaci and Ganea^30^). We do not question the notion that engagement matters. Rather, we simply note that our data provides no support for the notion that active human-centred learning is necessary for effective engagement.

We found no evidence that teacher effectiveness was conditioned on variables such as their understanding and acceptance of evolution (although these correlate), religiousness, highest biology qualification, formal evolution education, gender or the number of times they used the resources. In contrast, that the degree of improved teachers’ confidence was a repeatable predictor of student improvement points to the need for enhanced teacher training, particularly for non-specialist primary school teachers teaching a new topic of evolution.

Qualitative evidence from this study suggest that teachers found the support package helpful and perceived their understanding of the topic was improved by the intervention. To widen access to evolution education, we offer a free Massive Open Online Course (MOOC), *Understanding and Teaching Evolution*, designed for teachers and 14–16-year-old students, created in collaboration with the Darwin Correspondence project and the Galapagos Conservation Trust. (https://www.futurelearn.com/courses/understanding-evolution). All schemes of work can also be freely downloaded: (https://people.bath.ac.uk/bssldh/LaurenceDHurst/Outreach.html).

## Methods

### Development of the student questionnaire from existing assessment items

In order to collect large-scale quantitative data a simple paper and pencil multiple choice assessment instrument was developed. Based on the research of Flanagan and Roseman^31^ assessment items from the AAAS science assessment website were chosen for their appropriate cognitive demand and relevance to the UK National Curriculum. To allow the assessment of the concept as whole 15 assessment items were selected from 5 broad categories: common ancestry/homology, natural selection, variation, fossils/geological time and extinction, each item having four alternative answers with common alternative conceptions acting as distractors.

The original tool was field-tested across a broad range of demographics in 43 US states from grades 6–12 (11–18 years old). Therefore, the selected assessment items had to be adapted to reduce reading difficulty and cognitive load making them suitable for primary and middle school children in the UK. This was achieved by using diagrams and tables of comparison rather than large blocks of text together with reducing the length and complexity of sentences.

During the pilot phase, the students read and completed the full written amended version of the questionnaire individually. This format was found to take too long (~30–45 min) and concerns were also raised about reading speed and concentration spans. After consultation, the mode of delivery was altered to one in which the students marked their responses on a grid as the teacher read out the questions to the whole class. They were able to look at the question on the white board and given ample time to think about their answers. To ensure consistency across different schools, the teachers were directed to read each question in full and then summarize it by focusing on the key differences shown by emboldened text. The new mode of delivery was much quicker to complete (~20 min) and had the added benefits of reducing question fatigue and problems associated with poor reading skills. The final student assessment instrument is provided (Supplementary Notes 9 and 10).

### Mode of repeat testing

The students were assessed using the same test at three different time points: pre-teaching, as soon as possible after teaching (in practice around a week later in the next science lesson) and if possible 3–6 months later to evaluate long term retention. We opted to use the same question given information from the pilot phase. Notably, if a similar question is open to misinterpretation then its usage adds an unnecessary noise variable to the analysis. From the pilot project, it was clear that very subtle changes in language or in presentation of the questions could lead to misinterpretation (Supplementary Note 11). To minimize the possibility of a similar question not being as “similar” as intended, it was optimal to ask the same questions in the actual assessments.

Pre-testing and post-testing using the same assessment instrument is a commonly used method to assess student learning gains^32^, however, some studies have shown evidence of positive testing effect, i.e. taking a test leads to an improvement in learning compared with studying alone^33^. The relevance to our study is not so clear, however. Crucially, these experiments have involved testing the recognition or recall of specific, targeted pieces of information contained within the study materials, rather than the assessment of learning outcomes from a programme of instruction, the latter being what we did. Indeed, despite the large body of experimental memory testing evidence, only a few have been carried out within an educational context to investigate the positive effects of testing. Further, leaving aside the problem that the majority of studies involve much older students (usually undergraduates) in laboratory settings, the time periods between the initial and final test are usually relatively short, typically 1–2 days. Only a few consider the effects over longer time frames of up to 1 month, the sort of period over which our tests were performed.

While we don’t consider a positive testing effect to a priori be an important issue in our study, several devices were employed to mitigate any effect. First, the content of each assessment item within this instrument did not feature in any of the teaching intervention materials contained within the four different SoW, thus limiting student exposure to this material to the tests alone. This allowed the quantification of gains in learning outcomes or overall understanding from the sequence of lessons to be measured rather than recall or recognition of target material. This differed from the studies of testing reinforcement in which the opposite was true. Second, due to the mode of test delivery students only had access to the assessment items on a screen for as short a time as reasonable, minimising exposure to this material within each testing period. Third, and importantly, no feedback was provided during the study as to the correct answers. The students were, therefore, unable to correct errors in their understanding or confirm correct responses. With feedback important after multiple-choice question tests to prevent students repeatedly giving the same incorrect answers^34^, lack of feedback should have no augmentation. Additionally, participating teachers were not given the correct answers to the student assessment instrument to avoid any unconscious bias when they read out the questions.

If our method avoids positive reinforcement, it may end up being conservative as it could cause the opposite effect, namely negative suggestion, an increased belief in incorrect information that the students acquire through testing, not least because the incorrect answers were common alternative conceptions acting as attractive lures. Taking tests without feedback can give a slightly higher proportion of incorrect lure responses on the final multiple-choice question test compared to not taking a test. This is thought to be owing to a tendency for uncorrected errors to be especially likely to persevere. It usually takes several tests and feedback cycles to overcome this tendency to repeat errors. Similarly, the act of encountering false statements, even when students know they are false, can make them seem true at a later time. This mere-truth effect would also reduce the positive effects of testing. Indeed, Kelley and Lindsay^35^ found that undergraduate students could recall previously selected incorrect multiple-choice question lures more easily, this retrieval ease being misinterpreted as confidence in the correct answer. As all subjects were treated equally in our tests, negative suggestion is unlikely to explain variation between pupils.

### Collection of demographic and related data

Student demographic data was confined to name, gender and date of birth. In order to avoid confidentiality issues associated with disclosing formal science attainment scores, teachers were asked to give their judgement of the relative science ability of each student within their class as being either high (top 1/3 of class), middle or low (bottom 1/3 of class). To avoid requesting confidential personal information, composite demographic data were taken from the school’s most recent Ofsted report and Indices of Deprivation for use in the school-level analyses.

### Covariate considerations

While teaching-mode may make a difference, several other student-focused parameters might also predict variation in performance and potentially interfere with interpretation of results if randomization has failed. We consider three such parameters: biological sex, teacher-informed assessment of ability and student age. Some evidence suggests that primary school students think of science as a masculine subject^36^. According to this stereotype, boys are better at science than girls, which is often the implicit message in media headlines, leading to girls having a less positive attitude towards science and a reduced perception of their own abilities. However, meta-analysis^37^ reports comparable gender performance in UK primary school students, supporting the gender similarities hypothesis^38^. Alternatively, it might be the case that the trend for girls outperforming boys at aged 16 (GCSE in the UK)^39^ is emergent in primary schools.

Prior evidence in secondary schools suggests that aptitude/ability is a robust predictor of the response to the teaching of evolution in the UK context^40^. It might similarly be expected that students identified by their teachers as having higher “ability” in science relative to their peers would achieve a larger increase in marks after instruction.

Finally, it has been suggested that older students may be cognitively more able to grasp this difficult topic, particularly the more abstract concepts such as homology^18^. Amongst our pupils there is relatively little age variance as they are all within two school years. As a consequence, we don’t expect a strong effect, if any. We present multivariate analysis of student change in understanding (pre to post) incorporating these three variables along with teaching mode (i.e. SoW).

Aside from the lack of sequential testing, Leach and Scott^11^ also highlight several further issues that we considered in designing the implementation. In particular, when evaluating the effectiveness of one scheme over the other, they emphasise: (1) the need to have comparable student populations or be able to control for differences; (2) that the assessment instrument must not be biased towards one approach over the other; (3) that the results must be interpreted in light of teaching time provided and other costs and 4) the need to consider the role of the teacher in promoting student learning.

The first issue was dealt with by forming a large-scale randomised control with replication, along with covariate analysis. Note that test and replicate populations were well matched for age, gender and prior understanding (Supplementary Table 4). Regarding the second issue, the quantitative tool was designed not to be biased towards one SoW or the other, but as bias is often invisible the extent to which any such bias has been eliminated is impossible to know. By restricting to four lessons we rendered teaching time unusually uniform. Regarding costs, we were aware that expensive resources could bias results. To eliminate this variable for the trial, we supplied the resources. Nonetheless, cognisant that for adoption of any SoW the cost is a key issue (at least in the UK context), the schemes of work were designed to be minimal in cost. We estimate that the four lesson plans cost ~£0.10 per student (approx. $0.13), making them extremely cheap.

### Teachers as a variable

The fourth concern, teacher effects, are, by their very nature, harder to control in an experiment with in situ testing. This is potentially problematic as effective teaching can only succeed with capable teachers with a good understanding of evolution and a firm grasp of its unifying role within biology. Indeed, to minimize their students’ alternative conceptions teachers need to have an understanding of the subject matter together with appropriate pedagogical skills and specific Pedagogical Content Knowledge (PCK) related to those concepts. Problematically, whilst the development of scientific subject matter has been a required part of teacher training since the introduction of the UK’s National Curriculum, there are still many primary school teachers with relatively little science knowledge. Indeed, in our context, fewer primary school teachers are comfortable teaching evolution than secondary ones. Murphy, Neil and Beggs^41^ indeed report that around 50% of primary school teachers had a self-identified lack of confidence.

These teacher-centred issues present both an opportunity and challenge. It is an opportunity as, from our initial enquiries, teachers were very willing to engage with training and to adopt and stick to set lesson plans. Thus, in order to collect the large-scale data needed for our statistical analyses, an in situ programme of teacher training was carried out in all participating schools. This was both to improve the standard of evolution teaching and, more particularly, to facilitate standardised delivery of the SoW using the resources provided. As we were cognisant that even these attempts to force uniformity could never be claimed to be perfect, we also assessed teacher understanding and receptivity. This enables us to evaluate teacher-centric parameters as possible causes of differences in student gain in understanding.

In particular we were concerned that, despite the uniformity of the SoW and other teacher training, teacher-derived variation may yet persist, not least because possibly subtle differences in the way in which they communicate the topic can affect the way in which students judge the trustworthiness of the information. Sanders^42^, for example, found that some teachers who were sceptical about evolution somehow communicated this to their students. Less subtly, several studies have reported teachers presenting evolution as “only a theory”^43^. This lack of understanding as to what constitutes scientific evidence, and the misunderstanding of the tentative nature of science, were also addressed during the pre-teaching training sessions. Moreover, by assessing teacher acceptance of evolution we are able to factor this hard to control variable into our analysis as a predictor variable.

Even if the teachers were uniform in their acceptance of evolution, teacher-centred effects would not be fully removed as teacher confidence remains a potentially important variable. Many teachers experience problems translating guidance from reform documents such as the National Curriculum into the classroom, making it harder for them to understand how to organise the topic, divide it up appropriately and frame it for teaching. Additionally, when teaching outside of their “comfort zone” some teachers may act more like novice teachers during the interactive phase of the lesson, directing their classes to ‘safer’ activities. They may be more likely to devote more time to teacher talk, use resources such as worksheets and limit the opportunity for students to ask questions. While our teacher aides were intended to enable confident teaching, it is inevitable that the teachers’ different backgrounds will provide scope for different abilities, or perceived abilities, in teaching the material. We thus also evaluate teacher understanding as a covariate for the analysis. In addition, as teachers and students are embedded in schools that themselves are highly diverse, we also treat numerous school-level variables as possible explanatory variables.

We note, in addition, that the teacher training that we enabled to provide a more uniform base is not possible in the future for all primary school teachers. To this end, we make available our teacher resource packs along with the SoW (https://people.bath.ac.uk/bssldh/LaurenceDHurst/Outreach.html) and have developed a teacher-centred Massive Open Online Course (Understanding and Teaching Evolution, available for free on the Future Learn platform https://www.futurelearn.com/courses/understanding-evolution).

### Hypotheses

As required of medical CONSORT declarations, we formalise our expectations into null and active hypotheses. Specifically:

#### Effectiveness of teaching

H_0_: Teaching using the schemes of work results in no improvement in student understanding.

H_1_: Teaching using the schemes of work results in positive improvement in student understanding.

#### Effectiveness of individual schemes of work

H_0_: There is no heterogeneity between the four schemes of work in student gain in understanding.

H_1_: There is heterogeneity between the four schemes of work in student gain in understanding.

H_1.1_ Subsidiary hypothesis: there is heterogeneity between the four schemes of work in student gain in understanding with the rank order of effect sizes as provided in Table 2.

#### Interaction effects

H_0_: The outcome of each scheme will be predictable from knowledge of the effectiveness of subcomponents when these subcomponents are considered in isolation.

H_1_: The outcome of each scheme will not be predictable from knowledge of the effectiveness of subcomponents when these are considered in isolation.

### Development of teaching resources based on existing educational studies

**Fig. 8:**
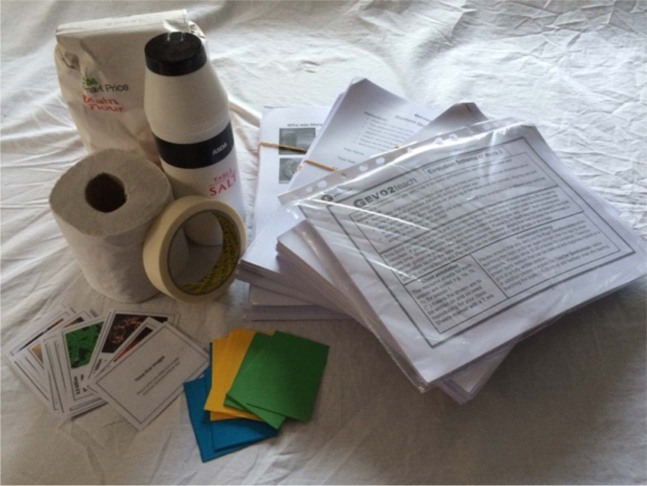
Example of resource package provided for use in participating schools. The full cost of the materials for each SoW was under £10 per class, including printing and lamination.

Detailed teaching SoW and resources for non-science specialist primary school teachers were based on the limited number of small-scale studies. The resources were adapted by liaising with partner trial schools leading to improvements in “teachability”. All resources developed for the project were fully differentiated and adjusted to be of the correct reading age, with a view to suitability for children of all abilities in mainstream schools. They were developed to cover the relevant parts of the Key Stage 2 National Curriculum and to support a scientifically valid understanding of evolution whilst avoiding emotional or religious conflict. The cost of the activities was minimized and used equipment that was easily available and suitable for classroom use. Participating schools received all materials so that there was no cost implication or additional preparation time (Fig. 8) rendering implementation uniform. For those utilizing the resources outside of the study, the costs are very low.

The SoW were developed to build upon pertinent biological concepts introduced earlier in their primary education. Students had previously learned about fossils on two occasions—in the year 3 rocks topic *‘Describe in simple terms how fossils are formed when things that have lived are trapped within rock’* and in years 4 and 5 through the topic of living things and their habitats, gaining an awareness of the variety of living things, classification, and how living things are adapted to their different habitats and interact together. The teaching intervention programmes were designed to cover the following aspects of the revised year 6 Key Stage 2 Programme of Study^44^:

- recognise that living things have changed over time and that fossils provide information about living things that inhabited the Earth millions of years ago
- recognise that living things produce offspring of the same kind, but normally offspring vary and are not identical to their parents
- identify how animals and plants are adapted to suit their environment in different ways and that adaptation may lead to evolution

The lessons were structured in line with Piaget’s model of cognitive development following the 3E learning cycle of Exploration, Explanation and Expansion. Each lesson consisted of three separate components: starter, main and plenary (the standard school lesson format used in schools). The main or work phase activities for Lesson 1 and Lesson 3 were identical in each teaching sequence. There were 2 alternative work phase activities for Lessons 2 and 4. Collectively this gave a total of four different pathways through the teaching materials, giving rise to the four different SoW. The content of the starter and plenary activities were novel or adapted from pre-existing teaching resources and were identical in each SoW. This arrangement ensured that the different work phase activities were embedded within the same conceptual framework and allowed the impact of the work phase activities of Lessons 2 and 4 to be evaluated separately and for possible interactions within lesson sequences to be identified. See Table 1 for a summary of the different work phase activities.

**Fig. 9:**
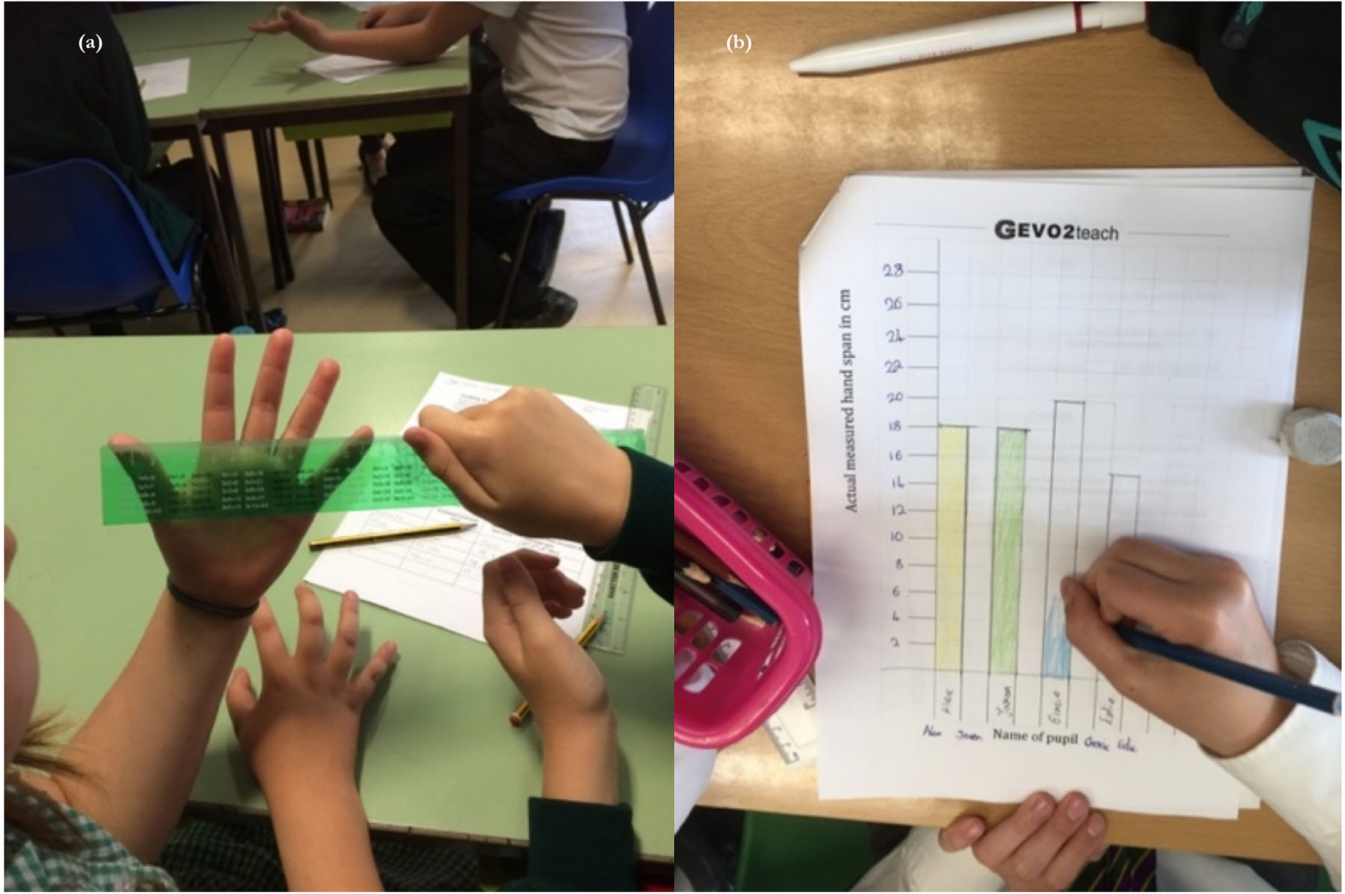
Photographs showing the main activity from Lesson 1, as example of intraspecific variation within the class. **a** Estimating and measuring; **b** transformation of the data into bar charts.

The work phase activities for Lessons 1 and 3 were based on suggestions from other researchers in the field (see below for details). The work phase activity of the first lesson (Fig. 9) of each teaching intervention was to introduce the existence of variation among individuals and to highlight intraspecific variation, a prerequisite for the correct mechanistic understanding of natural selection. Intraspecific data were collected in class and then mathematically processed in order give a wider appreciation of the variability within a species and help to overcome essentialist thinking.

The scale of geological time was introduced in Lesson 3 using a version of the “toilet roll of time”, adapted to cover 15 different significant events in the history of life on earth. This activity taught deep time in the way suggested by Catley and Novick^45^, who advocated providing students with knowledge of the correct timing of a small number of critical events together with the visualisation of the relative spacing of these events to provide a framework for greater understanding of evolutionary processes. The activities used in this lesson were developed with permission from existing teaching resources.

**Fig. 10:**
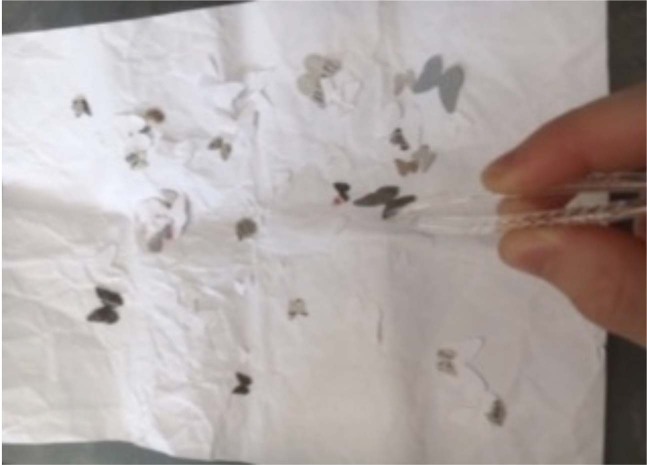
A photograph showing a student hunting paper moths in the student-centred hunting investigation forming one alternative activities for Lesson 2.

The main activities for Lessons 2 and 4 were based on existing age-appropriate educational resources. In Lesson 2, the peppered moth (*Biston betularia*) was chosen as a well-known exemplar of a species showing natural selection in action^46^. Two alternative activities were developed around the predation of peppered moths by birds to establish whether a “hands-on” student centred “moth hunting” activity was more effective than a seemingly more traditional teacher-centred PowerPoint activity. The “hunting” activity (Fig. 10) was based on the suggestion of Campos and Sá-Pinto^47^, with the students acting as the bird predators and using their forceps as beaks to “hunt” the moths on either white or newspaper background environments. Several rounds of timed predation followed by reproduction of the surviving moths were carried out to show differential survival and increased proportion of mimetic colours. During the activity students were asked to make and test predictions and then to explain the process.

**Fig. 11:**
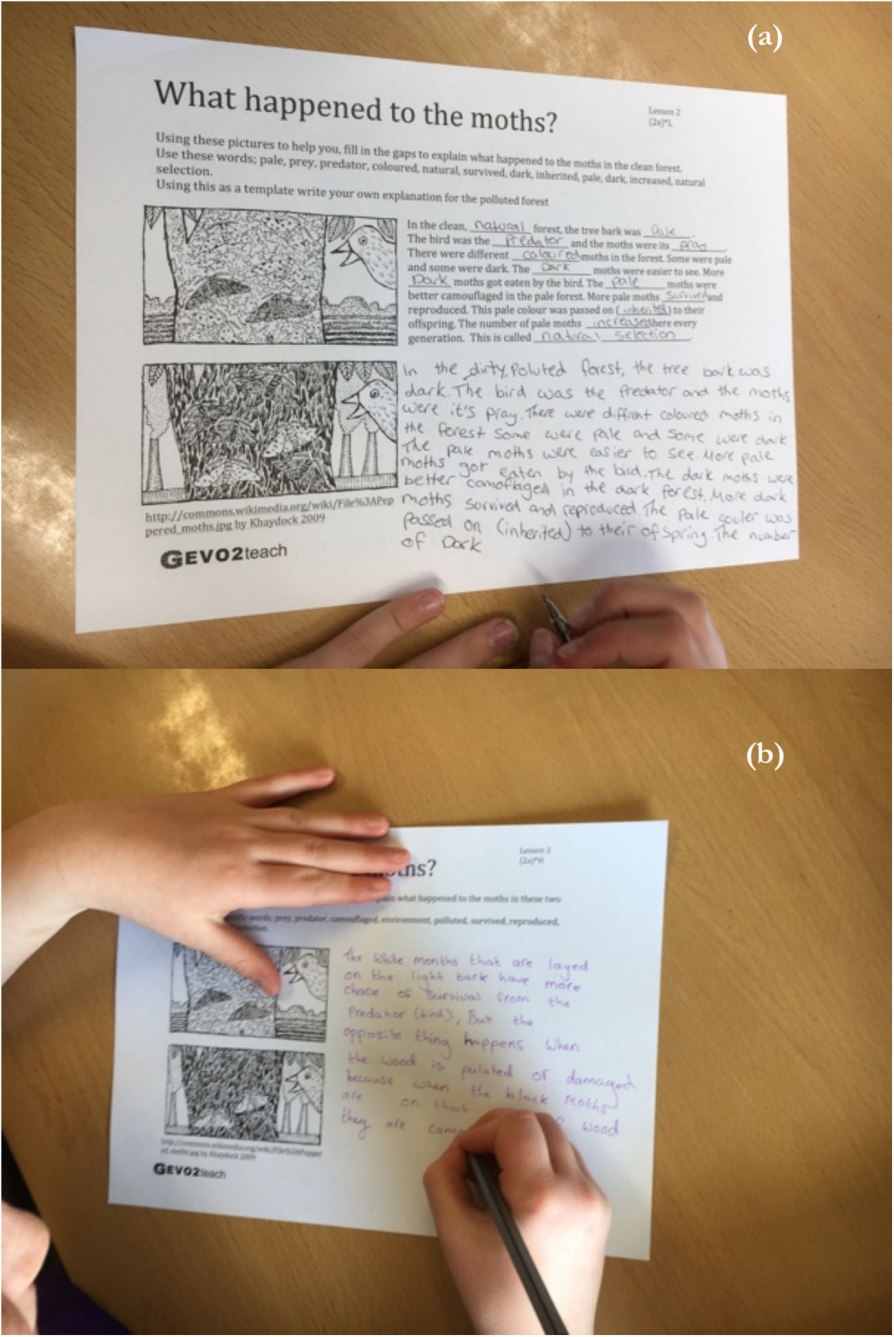
Photographs showing the nature of the differentiated written exercise carried out after the PowerPoint presentation forming the alternative moth main activity for Lesson 2. **a** Lower ability students were supplied with keywords and a cloze passage scaffold to explain the first diagram; **b** higher ability students were only supplied with the keywords with which to explain the diagrams.

The alternative moth activity was structured similarly to the picture story-book intervention of Kelemen, Emmons, Seston Schillaci and Ganea^30^. Their book was based on *pilosas* (a fictional species) and was shown to improve the understanding of natural selection in five to eight-year-old American children. Our PowerPoint presentation explained the process of natural selection in peppered moths and mirrored the pages of this story-book. After the presentation students were asked to explain the process of natural selection in their own words whilst provided with diagrams as visual stimuli and a glossary of terms. This scaffolded sheet was differentiated so that the students could decide their own level of difficulty (Fig. 11).

**Fig. 12:**
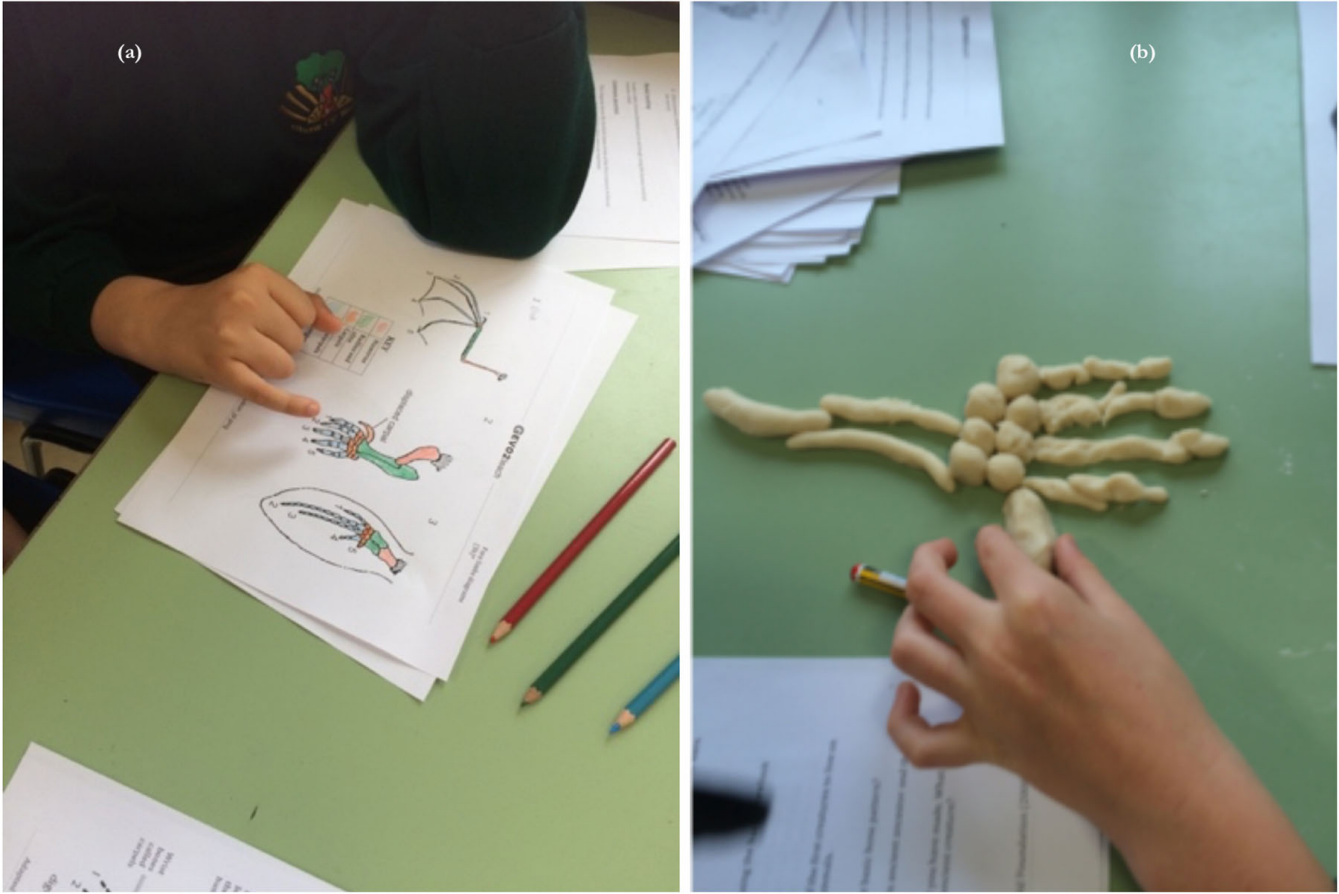
Photographs showing the extant example of homology and common ancestry activity from lesson 4. **a** identifying homologous bones in various tetrapod species; **b** salt dough modelling of a human arm.

**Fig. 13:**
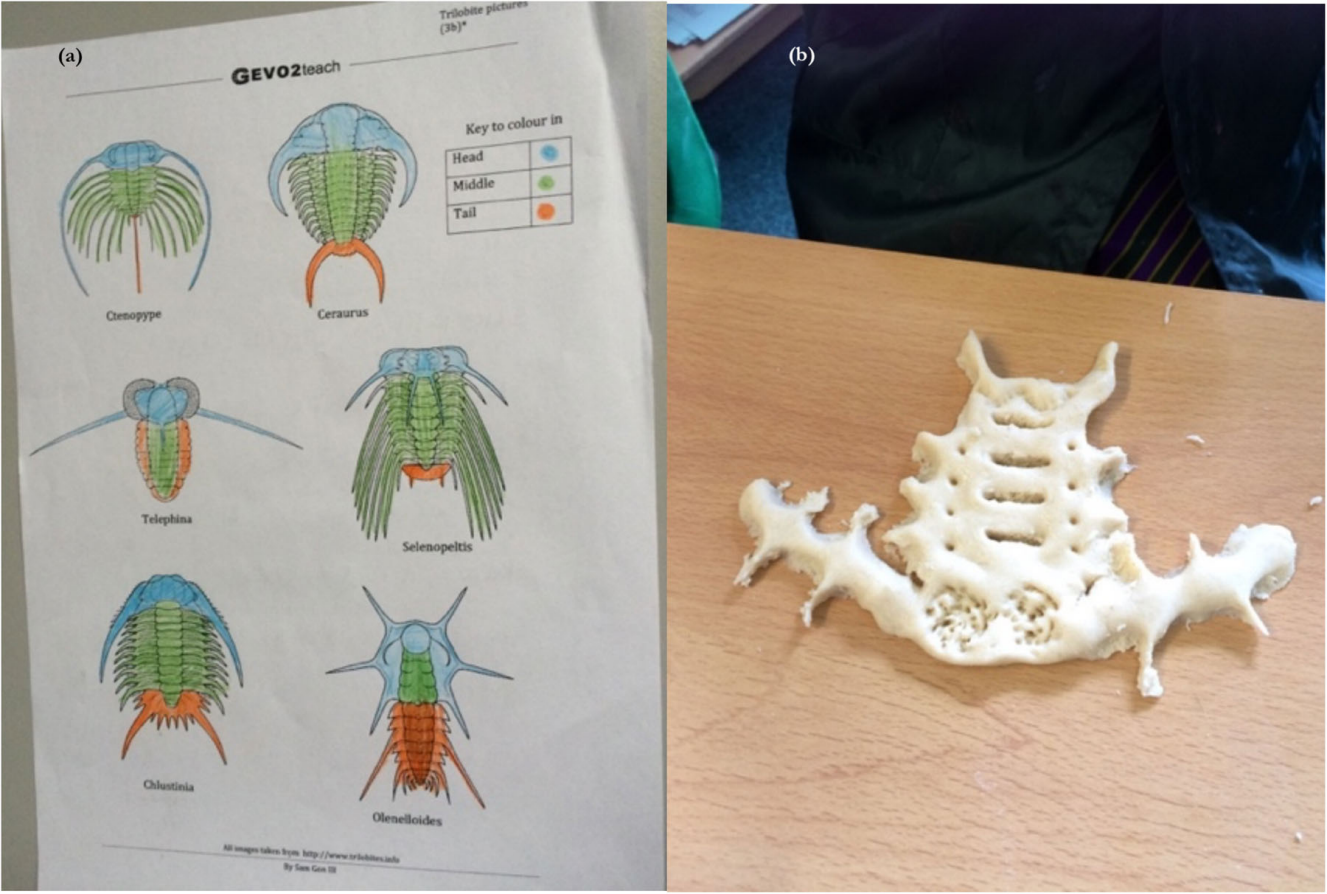
Photographs showing the extinct example of homology and common ancestry activity from lesson 4. **a** identifying homologous body parts in various Trilobite species; **b** salt dough modelling of a novel Trilobite.

The two alternative activities for Lesson 4 both involved the same learning experiences but were developed to establish whether homology and common ancestry were easier to understand if based upon either extinct species or us and our relatives. Both activities involved identification of homologous structures via salt dough model making. The structure of the pentadactyl limb (Fig. 12) in a range of tetrapod vertebrate organisms, including humans formed the basis of the extant example. This extant activity was based on the similarities/differences lesson of Nadelson, et al.^48^ which explored the identification of extant homologous structures by American children between 5 and 7 years old. Using work sheets adapted from existing resources from the Nuffield Foundation^49^ students in this current study identified homologous bones in a range of mammalian examples and then went onto model a human forelimb based on the patterns identified during the lesson. Various diverse Trilobites species adapted from Wagler^50^ were chosen to form the extinct example (Fig. 13). Table 10 provides a summary of how the main activities were embedded into the four lessons.

**Table 10:** Summary of the range of different activities into which the main activities were embedded.

Lesson 1	Variation
Starter	PowerPoint presentation on variation (definition, examples and causes) and continuum activity to show variation within the class.
Main	Quantitative investigation of variation within the class (eye colour, chin dimples and hand span) including mathematical processing of data and transformation into graphs.
Plenary	Hospital baby switch scenario exercise to explore understanding of the cause of variation in traits.
Lesson 2	Natural selection
Starter	PowerPoint presentation on adaptations (polar bear and cactus) and blob game to explore ideas of natural selection, selective advantage, differential survival and extinction.
Main	One of two alternative activities based on peppered moth populations as an example of natural selection. Either: (a) Student-centred “hunting” moths activity carried out in small groups or, (b) Teacher-centred activity based around a PowerPoint presentation and scaffolded written task explaining survival of mimetic peppered moths.
Plenary	Story board activity to show process of natural selection in an island dwelling bird population.
Lesson 3	Geological time
Starter	Video introducing concept that all organisms are related to a common ancestor, using whales as an example.
Main	Toilet roll of time activity using 200 squares of toilet roll to represent 4.6 billion years. Students order and position different cards along the timeline to show major geological events, from the formation of the earth to the present day, emphasizing their relationship to the same common ancestor.
Plenary	Mini spiral timeline to hang in class.
Lesson 4	Homology and common ancestry
Starter	PowerPoint presentation on how organisms have changed over time and are related to each other.
Main	One of two alternative activities both based around the identification of homologous structures within related species and model making. Either: (a) extant species focusing on the pentadactyl limb in tetrapods, or (b) extinct species focusing on Trilobites.
Plenary	Exercise on phylogenetic trees and the common ancestry of humans.

Three homework/extended reading exercises were also developed to raise awareness of the importance of the contributions made by historical figures (Mary Anning, Jean-Baptise Lamarck and Charles Darwin) to current evolutionary theory. The homework exercises were designed to improve comprehension of the chosen passages as well as numeracy and literacy skills. Completion of the homework was voluntary to match their status in the supplemental information section in the National Curriculum and the fact that not all primary schools set science homework or any homework at all. As this content was optional, care was taken to exclude these topics from any of the assessment items in the student questionnaire.

### Pilot phase

The teaching materials and student assessment instrument developed by this study were trialled in two primary schools during the summer of 2015. The purpose of the trials was to assess (a) whether the planned activities were both age-appropriate and logistically possible within a classroom setting and (b) whether the method of assessment was valid. Pre-instruction training was given to the normal classroom teacher, who was then observed by the principle researcher using the resources provided. Pilot school 1 (*N* = 16) was issued with SoW 1, whilst pilot school 2 (*N* = 30) was issued SoW 4, allowing all of the variable activities to be scrutinised. Notes were taken during the observations, including suggestions for improvement made by the pilot teachers. Where appropriate, the resources and assessment instrument were adapted to enhance the learning experience for the students and ease of use by the teachers. Statistical analyses of both sets of pre and post test results were also carried out to confirm the validity of the assessment method before being used to collect quantitative data in the live experiment. Although the primary school teachers were non-specialists they were experienced classroom practitioners. This input from primary school teachers and improvement in “teachability” ensured that the post-pilot resources described in the previous section had widespread support from the primary community.

### School recruitment

All primary and middle schools within a 50-mile radius of the Bath (Somerset, UK) were invited to participate in the study. This distance allowed for individual face-to-face contact and teacher training in 45 different schools recruited across the Southwest containing a mixture of rural and urban locations. For confidentiall reasons the identities of the schools cannot be disclosed.

Recruitment strategies included phone calls, letters, emails and promotional postcards to, when possible, named teachers within the schools. A ~10% uptake in schools contacted was achieved, together with a school completion rate of 90%, the latter being unusually high in our experience. For a summary of student participation for both Tranche 1 (2016–2017) and Tranche 2 (2016–2018) see Table 11. Tranche 2 was collected over two academic years. All schools in the study did not select students on the basis of ability. Only one school in either tranche was an independent school, the rest being state schools.

**Table 11:** Summary of participation in the two tranches.

Attribute	Tranche 1 (2016–2017)	Tranche 2 (2016–2018)
Number of schools	17	28
Number of classes	40	56
Number of teachers	37	46
Number of students completing pre-test	1152	1505
Number of students completing pre and post-test	988	1309
Number of students completing the pre, post and retention tests	320	523
Number of primary schools	9	24
Number of middle schools	8	4

### Allocation of schemes of work

One of the four SoW was allocated by the principal researcher (LB) without any input from the school. Allocation was not fully randomised but, given the limited number of schools, was done in such a way as to a mix of primary and middle schools, locations and approximately equal numbers of students in the four SoW (Table 12). Allocation was random in that it was done blind to covariates (e.g. socio-economic status, Ofsted rating). In schools participating in both tranches, the “opposite” SoW was allocated in Tranche 2 so they delivered both alternative main activities for Lessons 2 and 4.

**Table 12:** Breakdown of participation by SoW of the 2297 students who completed both the pre and post teaching tests.

Attribute	Scheme of work allocated			
	1	2	3	4
Number of students	593	602	519	583
Number of primary schools	9	8	8	8
Number of middle schools	3	2	4	3

### Teacher training

After allocation of a randomly allocated SoW, a mutually convenient time slot of approximately one hour was arranged with the school for the principal researcher (LB) to deliver the resources and conduct standardised teacher training. This was done to prevent interference with teaching (i.e. at lunch time, free time within the school day or after school). In order to ensure all teachers understood how to correctly use the resources and carry out the activities, the SoW allocated to each teacher was scrutinised lesson by lesson with each individual activity discussed and demonstrated. Guidance was given on how to schedule the lessons to fit into their allocated science lessons, classroom management, the appropriateness of the differentiated tasks and how to overcome potential behavioural/logistical/religious problems. Any common alternative conceptions that their students might hold, as well as any questions that arose, were discussed with reference to the activities provided in the resource packs. This programme of standardised teacher training, detailed teacher information sheets and mark schemes were provided to minimize the possibility of unwanted or uncontrolled between-teacher heterogeneity.

### Feedback on the utility of the allocated resources

Qualitative feedback from all participating teachers was obtained after collection of the questionnaires at the end of the topic during a prearranged, mutually convenient, time slot of at least one hour. Individual or small group interviews were carried out in all participating schools with more than one participating teacher to allow for personal perspective. Data were collected from field notes and around 60 h semi-structured interviews. These were audio taped and transcribed verbatim to enhance the understanding of statistical enquiry. Systematic, thematic emergent coding was carried out in order that this qualitative data could be included in the study. This was to enable a deeper exploration of the research questions by encompassing both objective and subjective standpoints, adding to the richness of the findings.

### Feedback on the questionnaire and resources from student focus groups

Qualitative feedback from a representative sample of students was obtained after collection of the questionnaires at the end of the topic. The focus group interviews were conducted with small groups of students who had been withdrawn from their classes. The semi-structured interviews focused on the student questionnaire, its format, readability, difficulty and whether they understood what the questions meant as well as the resources they remembered. The interviews were audio taped and then transcribed verbatim.

### Input error checking

As all of the quantitative data was entered by the principal researcher, analysis of inter-rater reliance was not needed. However, input error checking was carried out independently by a colleague on a representative random sample of 50 student questionnaires (pre, post and retention). When the entered data were compared, 18/900 (2%) were incorrectly entered, however only 0.6% of the errors affected student score for the 15 assessment items, the others being due to incorrect transcriptions.

### Statistics

The data sets generated and analysed during the current study together with the scripts which were implemented in R and are available in the repository [https://github.com/edmllb/GEVO2teach]. Where standard statistics are employed, we note these in text. Two particular methods are employed that we describe here in further detail.

First, we consider LOESS regressions. To allow for ceiling effects and to normalize data to heterogeneity in pre-testing scores, we employ a method of locally weighted polynomial regression (LOESS) previously employed by Mead, Hejmadi and Hurst^40^. For each relevant student, we determined the difference between their pre and post teaching scores. This difference was then modelled with the pre-teaching scores using the LOESS function in R. We then extracted the residuals for each data point (student) from this model output. Thus, the residuals provide the relative change in student performance given prior performance.

Second, we consider interaction effects. To estimate the extent of interaction terms we employ the method of Sevdalis and Jacklin^21^ derived for 2 × 2 designs (employed for drug interaction studies). We performed a LOESS regression as described above. From this, the mean LOESS residual for each SoW were calculated and assigned to a 2 × 2 table (Supplementary Table 5) such that the mean residual for each SoW corresponded to the unique combination of Lesson 2 and 4 performed. An activity mean was calculated for each activity (i.e. the hunting moth activity mean was the mean of the mean residual from SoW 1 and SoW 2) and a grand mean calculated by taking the mean of all mean residuals for all SoW. The interaction effect for each SoW was then estimated as per Sevdalis and Jacklin^21^ using the formula: Interaction = Mean SoW residual – grand mean – corresponding Lesson 2 activity mean residual − corresponding Lesson 4 activity mean residual. For example, the SoW 1 interaction = Mean SoW 1 residual – grand mean – hunting moths activity mean residual – trilobites activity mean residual. The summed absolute interaction effect was calculated by summing the absolute interaction effect for each SoW.

In order to establish whether interaction effects differed significantly from what might be expected due to chance alone, we randomly shuffled the SoW assigned to each student and recalculated the interaction effects as above. This was repeated for 10,000 simulations, providing a null distribution of interaction effects. From these simulants, we calculated a one-tailed empirical P-value using the formula *P* = *m* + 1 / *n* + 1 where *m* = the number of simulants with an interaction effect greater of equal to the true interaction effect and *n* = total number of simulants. For negative interaction effects, the one-tailed test was calculated in the opposite direction.

### Consort declarations

Funding: The project was funded by the Evolution Education Trust (EET). No grant number specified. The funder had no role in the design, implementation, analysis or interpretation of the study.

Design pre-specification: The design of the project was specified in the grant to the EET, available from EET.

Sample size: sample size was not pre-specified. The sample was maximized given the time available.

Blinding: Owing to the nature of the intervention neither schools, nor teachers, nor students could be blind to their treatment.

Harms: We are unaware of any harms associated with the study.

Termination: The trial was terminated when funding was exhausted and with a view to work being written up. Late arriving responses were not included.

For CONSORT checklist, see Supplementary Table 6.

### Ethical considerations

Research with children presents special issues as young students are more vulnerable, have fewer legal rights and may not understand the language of informed consent. Appropriate ethical clearance for the project was provided by the Departmental Research Ethics Officer at the University of Bath by completing an EIRA (Ethical Implications of Research Activity) assessment prior to data collection. Legislation recognises that educational research that involves activities that are within the customary, usual procedures of schools and that involve little or no risk to the participants are exempt from the formal review processes. Additionally, the subjects were not deceived in any way during the study. For this reason, individual parental permission for the students to complete the student questionnaire was not requested. However, each school was provided with a written Plain English statement outlining the nature of the project before participation (Supplementary Note 12). The statement outlined the students’ right to privacy and was worded so that they could clearly understand the process in order to obtain their informed consent. Some schools also chose to publish this letter to parents to inform them of the study.

Prospective participating teachers and their students were informed in writing of the intentions behind the study and were notified that confidentiality and right to privacy would be maintained. They were also assured that there would be no harm to them as individuals and that the results of the study would not influence their grades or performance assessments within school. They were informed that participation in the study was voluntary and that they were free to opt out at any time during the process.

Written permission was obtained for all audio taped discussions from both teachers (Supplementary Note 13) and students as this was outside of normal classroom practices. A letter informing the parents and guardians of the students in the focus groups was accompanied by a consent form that parents were requested to complete if they approved of their participation in the focus groups (Supplementary Note 14). Only those students whose parents or guardians gave consent were included in this aspect of the study. In addition, individual permission was obtained from their teachers before their qualitative feedback was collected. Care was taken when taking photographs to exclude facial features; where faces do appear in this study, the schools in question held pre-existing written parental forms permitting these images to be shown.

### Reporting summary

Further information on research design is available in the Nature Research Reporting Summary linked to this article.

## Supplementary information

Supplementary Materials

Reporting Summary

## Data Availability

The data sets generated and analysed during the current study are available in the repository: https://github.com/edmllb/GEVO2teach.

## Code availability

The scripts which were implemented in R are available in the repository: https://github.com/edmllb/GEVO2teach.
