# Supplementary material for: A RCT for assessment of active human-centred learning finds teacher-centric non-human teaching of evolution optimal
Source: NPJ Sci Learn. 2020 Dec 4;5:19. doi: 10.1038/s41539-020-00078-0 (PMC7718267; doi:10.1038/s41539-020-00078-0)
Supplement: Supplementary file 1 — Supplementary Materials [file 41539_2020_78_MOESM1_ESM.pdf]

Supplementary Note 1: Validity and reliability of the assessment instrument

First, to assess internal reliability (alias consistency) we considered Cronbach's alpha (ranging from 0, no consistency, to 1). The pre-tests gave a raw alpha of "questionable" reliability in tranche 1 ($\alpha = 0.66$) and "acceptable" in tranche 2 ($\alpha = 0.73$). The coefficients improved in both tranches to an "acceptable" reliability ($\alpha = 0.71$) in tranche 1 and "good" ($\alpha = 0.8$) in the post-instruction tests. In both tranches the retention tests returned very similar values ($\alpha = 0.87$ and 0.88) indicating good consistency. We conclude that the test's internal reliability is adequate and can be replicated. The improvement in consistency after teaching is to be expected for a test of concepts that are unlikely to have been previously introduced to the students.

Second, under the null hypothesis that the assessment method was not fit for purpose (e.g. the students are guessing randomly), we would expect that the pre and post test scores would be uncorrelated. We found a significant positive Spearman's rank correlation in both tranches of data (tranche 1: $\rho = 0.44$, $P < 2.2x10^{-16}$; tranche 2: $\rho = 0.39$, $P < 2.2x10^{-16}$; see Supplementary Figure 1). Taken together with the combined P-value = $2.90x10^{-11}$ (Fisher's method), both tranches show moderate effect sizes from their correlation coefficients. The correlation in both tranches is not compatible with the null "random guess" hypothesis and shows good reliability between data sets.

The above test, could, however be explained if students simply present the same answer to any given questions in the pre and post testing phases. To address this, we consider student ability as a predictor. We would expect students identified by their teachers as having higher science ability to possess greater *a priori* knowledge of the topic and consequently achieve higher marks before formal instruction. A significant positive Spearman rank correlation with moderate effect size between teacher-assessed science ability and pre-test score is seen in both tranches (tranche 1: $\rho = 0.34$, $P < 2.2x10^{-16}$; tranche 2: $\rho = 0.33$, $P < 2.2x10^{-16}$) with a combined P value = $2.9x10^{-11}$ (Fisher's method). There was also a significant difference in the pre-test scores between all three ability groups in both tranches (tranche 1: $\chi 2 = 136.22$, P = 0, Kruskall-Wallis rank-sum test, post hoc Dunn test and Bonferroni correction; tranche 2: $\chi 2 = 161.33$, P = $< 2.2x10^{-16}$, Kruskall-Wallis rank-sum test, post hoc Dunn test and Bonferroni correction) both reporting moderate effect sizes (tranche 1: $\varepsilon^2 = 0.12$; tranche 2: $\varepsilon^2 = 0.11$). The means and medians were also in the expected direction: higher teacher-assessed science ability students achieved higher pre-teaching scores (see Supplementary Figure 2). These results indicate both that the students weren't guessing and that the test discriminates.

Fourth, the assessment instrument needs to be accessible and of the correct difficulty. "Readability" is of great importance as it allows students to access the information within an item and formulate their response. Using the assessment method developed by Kincaid et al. [1] the post-pilot adapted assessment items were easier to read, with a reading ease score = 70.04 and more appropriate reading grade level (5.88) for this age range compared with the original version (63.21 and 7.17 respectively). The assessment instrument was also of appropriate difficulty for this cohort of students with the mean percentage of correct answers increasing post instruction in both tranches (tranche 1: pre = 36.56%, post = 52.83% (+16.27%); tranche 2: pre = 38.14%, post = 53.91% (+15.77%)) with only a slight decrease in the retention test means (tranche 1: retention = 49.18% (-3.65%); tranche 2: retention = 53.52% (-0.39%)). The post instruction figures are close to the optimum difficulty score of 62.5% for this 4 options per assessment item Multiple Choice Questionnaire [2]. Our method relied on the use of circles and tables to specify specific answer responses rather than text. Our qualitative analyses supported their utility:

Students in the focus groups clearly expressed that they understood the use of circles (questions 1 and 3) and tables with ticks and crosses (questions 8, 10, 12 and 13), preferring them to the more descriptive text questions which some found too wordy and difficult to keep the different options in their minds.

- G1 [text-based question 9 is] *"Very long. For me, it's just like my eyes go everywhere on the page. Yeah, it's too much to focus on. But question 10 looks clearer than the other one as the table is much easier to read."*

- G2 "*I like those [circle] questions."*

- G3 "*I get lost and then I have to start again, 'cause I don't know where I was on the longer [text] questions."*

In addition, the assessment instrument must also allow clear discrimination between individual students. Item discrimination, the degree to which students with high overall test scores also got a particular item correct, was also investigated. In order to separate high and low ordered students, individual student test scores were used to obtain the total score of the cohort. Upper and lower groups were then defined as the top and bottom third of this total score. The discrimination index was then calculated using the formula; discrimination index = (number correct in the upper group - number correct in the lower group) / size of each group, and returned for each item ranging between 0.00 to 1.00. Larger values indicate that students who got any one item correct also achieved a relatively high score on the overall test, with a recommended item discrimination of at least 0.20. All items returned positive indices, with mean values in excess of 0.30, well above the 0.20 threshold in all tests in both tranches. In tranche 1, pretest, post test and retention test values were 0.31 ± 0.12, 0.34 ± 0.06 and 0.36 ± 0.12 respectively. In tranche 2 they were 0.33 ± 0.13, 0.40 ± 0.07 and 0.44 ± 0.10. This indicates that the assessment instrument was effective

at discriminating those students who understood the content from those who do not, rather than just guessing the correct answers.

Finally, student completion of the assessment items was considered as a function of item number to check for any evidence of question fatigue. The correlation between mean class percentage of null (NA) and ambiguous (U) responses and assessment item number was conducted using the combined data from both tranches of students who completed all three assessments (pre, post and retention) to allow direct comparisons to be drawn. Using one-tailed Pearson's correlation tests, no significant correlations between assessment item number and the mean class percentage of null (NA) or ambiguous (U) responses in either the pre or post assessment sessions were found. All correlations had small effect sizes ($R < 0.3$), except the post result for U responses which had a "moderate" effect size ($R = 0.43$). These results suggest that, although there were positive correlations between increasing assessment item number and the mean class percentage of NA and U responses in all assessment sessions, the effect of question fatigue was not a significant confounding factor in the pre and post assessment session results on which the majority of our analysis was focused.

A significant Pearson's correlation between assessment item number and the percentage of both NA ($R = 0.46$, $P = 4.20x10-2$) and U ($R = 0.47$, $P = 3.82x10-2$) responses was, however, found in both retention tests, with moderate effect sizes. The effect of question fatigue negatively impacting on the results of the retention test cannot thus be ruled out and could explain some of the waning effect encountered within individual assessment items, despite the percentage of NA/U responses being extremely low. In trache 1 the pre-test mean, pre-test median, post-test mean, post-test median and retention test mean and median were 1.21 ± 0.29, 1.16, 0.09 ± 0.11, 0.00, 0.23 ± 0.25 and 0.15 respectively. The corresponding figures for tranche 2 are 0.69 ± 0.38, 0.55, 0.18 ± 0.15, 0.15, 0.31 ± 0.20 and 0.25 respectively.

Importantly, low ability students responded well to teaching, with post test scores being higher than pre-test in both tranches ($P < 2.2x10^{-16}$, Wilcoxon signed rank test) with both effect sizes above the 0.4 threshold (tranche 1: $N = 205$, Cliff's $d = 0.60$, 95% CI: 0.51 – 0.67 (large); tranche 2: $N= 293$, Cliff's $d = 0.44$, 95% CI: 0.36 – 0.52 (moderate)). This suggests that resources developed by this project were appropriate for all abilities in mainstream schools.

1. Kincaid JP, Fishburne Jr RP, Rogers RL, Chissom BS. Derivation of new readability formulas (automated readability index, fog count and flesch reading ease formula) for navy enlisted personnel. DTIC Document, 1975.
2. Kaplan R, Saccuzzo D. Psychological testing: Principles, Applications and Issues (4e éd.). Pacific Grove: Brooks. Cole Publishing (1re éd. 1982); 1997.

Supplementary Note 2: Evidence for longer term retention

A common feature of many teaching interventions is that they lead to short term improvements in understanding which are subsequently lost. To address the issue of longer-term retention, the results obtained from students who took all three tests (although a more limited sample) were considered (tranche 1: n = 320; tranche 2: n = 523). The period between the post and the retention tests were similar for both tranches (tranche 1: mean 130.93 ± 73.20 days; tranche 2: 134.96 ± 64.78 days).

If there is some degree of longer-term retention, it would be expected that retention scores should be significantly higher than the pre-test scores. We find this to be the case (tranche 1: $P = 2.1 \times 10^{-14}$, Kruskal-Wallis rank-sum test, post hoc Nemenyi post hoc test; tranche 2: $P < 2.2 \times 10^{-16}$ in tranche 2, Kruskal-Wallis rank-sum test, Nemenyi post hoc test), with a combined P-value = 2.86×10^{-11} (Fisher's test). Second, if there is a waning effect, whereby over time any gains made are gradually lost, post teaching scores would be expected to be higher than retention scores. We found this to be the case for tranche 1 ($P = 3.60 \times 10^{-3}$, Kruskal-Wallis rank-sum test, Nemenyi post hoc test) but not for tranche 2 (P = 0.50, Kruskal-Wallis rank-sum test, Nemenyi post hoc test) (Supplementary Figure 3). However, these results give a statistically significant combined P-value ($P = 1.31 \times 10^{-2}$ Fisher's test), with both tranches showing moderate effect sizes (tranche 1: $\varepsilon^2 = 0.17$; tranche 2: $\varepsilon^2 = 0.13$). Results are consistent with the teaching interventions having some degree of long-term retention, however some of the understanding waned over time.

Supplementary Note 3: Use of LOESS residuals

We considered all data for which we have a pre and post test score for any given student, the relationship between the absolute change in score (defined as post score – pre-score) and the pre-test score. We then considered the residuals from a regression line as our metric of student given pre-test score. A regression was considered for the two tranches independently. To control for the possibility that standard linear regression's assumptions are not upheld, the residuals of a non-parametric LOESS curve for the change in student score against pre-test score were used.

The residuals may be considered as change in understanding given prior understanding and should have a mean near zero, indicating an average degree of change given pre-test score. This method also mitigates some of the "ceiling effect" problem present in any form of quantitative assessment, in which students with higher pre-test scores can only make smaller relative gains compared with lower scoring students as their initial score is already high. Likewise, students with lower initial scores have the potential to make larger relative gains but also smaller relative losses. Analysis of the uncorrected change in score with pre-test score confirming that this ceiling effect is pertinent to both data sets, there being a negative correlation of moderate effect size in both tranches between absolute change in score and pre-test score (tranche 1: $\varrho = -0.44$, $P < 2.2\text{x}10^{-16}$, Spearman's rank correlation; tranche 2: $\varrho = -0.40$, $P < 2.2\text{x}10^{-16}$, Spearman's rank correlation).

In addition, by using the residuals from the LOESS regression we can correct for the uneven distribution of raw pre-test scores between the four SoW (tranche 1: $\chi 2 = 16.27$, $P = 9.9\text{x}10^{-4}$, $N = 988$, Kruskal-Wallis rank-sum test, with Bonferroni correction; tranche 2: $\chi 2 = 20.59$, $P = 1.3\text{x}10^{-4}$, $N = 1309$, Kruskal-Wallis rank-sum test, with Bonferroni correction). While these results suggest heterogeneity between the four schemes as regards the pre-test scores (these results gave a combined $P = 2.1\text{x}10^{-6}$ (Fisher's test) across all 4 SoW), the effect sizes are small (tranche 1: adjusted r^2, $\varepsilon^2 = 1.70\text{x}10^{-2}$; tranche 2: $\varepsilon^2 = 1.60\text{x}10^{-2}$). Thus, we are confident that the residual method will eliminate this minor deviation from random.

Supplementary Note 4: Student level multivariate test

Ability, but not age or gender, repeatedly predicts gain in understanding

Using a multivariate model exploring the combined effects of gender, age, ability and SoW and find that we can account for 7.10% - 8.30% of the total variance in student score across both tranches of data (tranche 1: adjusted $R^2 = 0.071$, $P = 8.67 \times 10^{-15}$; tranche 2: adjusted $R^2 = 0.083$, $P < 2.2 \times 10^{-16}$). Results indicate that age was not significant (tranche 1: factor coefficient = -6.61×10^{-5}, P = 0.86; tranche 2: factor coefficient = -1.68×10^{-5}, P = 0.96), while students with higher science ability (as judged by their teachers) improved more, given their pre-test performance (tranche 1: factor coefficient = 0.48, $P = 7.93 \times 10^{-8}$; tranche 2: factor coefficient = 0.83, P = $< 2.2 \times 10^{-16}$).

Gender effects were weak and not replicable. In the multivariate analysis girls scored significantly higher than boys in tranche 1 (factor coefficient = 0.35, $P = 7.00 \times 10^{-3}$) but not significantly so in tranche 2 (coefficient = 0.24, $P = 6.40 \times 10^{-2}$). Student gender has only a weak effect size when analysed in isolation (tranche 1: Cliff's d = 0.09, 95% CI: 0.02 – 0.16; tranche 2: Cliff's d = 0.06, 95% CI: -4×10^{-3} – 0.12). This result underscores the results of large meta-analyses of gender effects on examination performance [1, 2] who caution against undue emphasis on statistical significance while effect sizes were ignored. Such a practice can exaggerate the importance of gender differences. It also supports the conclusion of the National Pupil Database [3] that reported negligible differences in student performance at KS2.

1. Wilkinson L. Statistical methods in psychology journals: Guidelines and explanations. American psychologist. 1999;54(8):594.
2. Hyde JS, Linn MC. Gender similarities in mathematics and science. Science. 2006;314(5799):599-600.
3. Department for Education. National curriculum assessments at key stage 2 in England, 2017 (revised). 2017.

Supplementary Note 5: Acceptance and understanding of evolution amongst primary school teachers weakly correlate

Whilst it is recognised that fewer primary school teachers are comfortable with teaching evolution [1], there are very few studies focusing on this group of teachers [2]. Multiple researchers have called for empirical work to be conducted to explore their understanding and acceptance of evolutionary theory to add to the limited number of studies in this area [3-6]. As the acceptance and knowledge of evolution are sufficiently different constructs they should both be examined when considering an individual's perspective on evolution [7, 8].

A positive correlation between teacher acceptance of evolutionary theory (as assessed by the MATE instrument [9]) and the understanding of natural selection (as assessed by using the CINS instrument [10]) was shown for both tranches (Supplementary Figure 4). The correlations for both tranches had similar moderate effect sizes, but only the correlation for tranche 2, with the greater number of participating teachers, was statistically significant (tranche 1: $\varrho = 0.33$, $P = 0.094$; tranche 2: $\varrho = 0.34$, $P = 0.025$, Spearman's rank correlation). However, a significant combined P-value ($P = 0.016$, Fisher's test) suggests that there is a significant positive correlation between teacher acceptance of evolution and the understanding of natural selection in this cohort. However, as observed in our prior analysis of secondary school students (Mead et al. [11] and elsewhere [12], this correlation is notably weak.

1. Fowler SR, Meisels GG. Florida teachers' attitudes about teaching evolution. The american biology Teacher. 2010;72(2):96-9.
2. Glaze AL, Goldston MJ. US Science Teaching and Learning of Evolution: A Critical Review of the Literature 2000–2014. Science Education. 2015;99(3):500-18.
3. Asghar A, Wiles JR, Alters B. Canadian pre-service elementary teachers' conceptions of biological evolution and evolution education. McGill Journal of Education. 2007;42(2):189-209.
4. Nadelson L. Preservice teacher understanding and vision of how to teach biological evolution. Evolution: Education and Outreach. 2009;2(3):490-504.
5. Nadelson L, Nadelson S. K-8 educators perceptions and preparedness for teaching evolution topics. Journal of Science Teacher Education. 2010;21(7):843-58.
6. van Dijk EM, Kattmann U. Teaching evolution with historical narratives. Evolution: Education and Outreach. 2009;2(3):479-89.
7. Shtulman A. Qualitative differences between naïve and scientific theories of evolution. Cognitive psychology. 2006;52(2):170-94.
8. Southerland SA, Sinatra GM, Matthews MR. Belief, knowledge, and science education. Educational Psychology Review. 2001;13(4):325-51.
9. Rutledge ML, Warden MA. The development and validation of the measure of acceptance of the theory of evolution instrument. School Science and Mathematics. 1999;99(1):13-8.

10. Anderson DL, Fisher KM, Norman GJ. Development and evaluation of the conceptual inventory of natural selection. Journal of research in science teaching. 2002;39(10):952-78.
11. Mead R, Hejmadi M, Hurst LD. Teaching genetics prior to teaching evolution improves evolution understanding but not acceptance. PLoS biology. 2017;15(5):e2002255.
12. Fiedler D, Sbeglia GC, Nehm RH, Harms U. How strongly does statistical reasoning influence knowledge and acceptance of evolution? Journal of Research in Science Teaching. 2019.
13. Rutledge ML. Indiana high school biology teachers and evolutionary theory: Acceptance and understanding. 1996.

Supplementary Note 6: Multivariate analyses excluding middle/primary schools

To check the validity of our findings at the student-level, we carried out additional student-level analyses by excluding all data obtained in middle schools and then similarly primary schools. As these analyses reduced the sample size, data from both tranches were combined before analysis. The results of this new multivariate analysis of predictors of loess residuals, now excluding middle school data (N = 1,249), were very similar to those of the complete tranche 2 data set (adjusted $R^2 = 6.03x10^{-2}$, $P = 7.03x10^{-16}$), confirming neither age (factor coefficient = $-4.08x10^{-5}$, P = 0.90) or student gender (factor coefficient = 0.95, P = 0.15) were significant factors. Additionally, LOESS residual scores increased significantly with increasing teacher assessment of student science ability (coefficient = 0.70, $P = 2.09x10^{-13}$). SoW 1 was also the least effective relative to the other SoW, however, it was only significantly less effective than SoW 2 (factor coefficient = 0.79, $P = 1.01x10^{-5}$) and SoW 3 (factor coefficient = 1.04, $P = 1.61x10^{-7}$) but not SoW 4 (factor coefficient = 0.36, P = 0.08).

When a second multivariate model was carried out with LOESS residual scores and the combined effects of gender, age, ability and SoW excluding data from all primary schools (N = 1048), we obtained similar results to those of tranche 1 (adjusted $R^2 = 7.1x10^{-2}$, $P = 9.76x10^{-16}$). Age was again found not to be significant (factor coefficient = $-6.00x10^{-4}$, P = 0.21), but boys scored significantly lower than girls (factor coefficient = -0.38, $P = 1.93x10^{-3}$). Additionally, LOESS residual scores increased significantly with increasing teacher assessment of student science ability (coefficient = 0.61, $P = 3.1x102^{-12}$). SoW 1 was also significantly less effective relative to the other three SoW; SoW 2 (coefficient = 0.78, $P = 7.66x10^{-5}$), SoW 3 (coefficient = 0.67, $P = 3.41x10^{-4}$) and SoW 4 (coefficient = 0.38, $P = 1.69x10^{-2}$). Interestingly, the similarity of these with prior results mirror the relative composition of the two tranches of data (see main text), for which there was a higher proportion of middle schools in tranche 1 when compared with a higher proportion of primary schools in tranche 2.

Supplementary Note 7: Teacher Endorsement Evidence

First, the feedback on lesson 2 was considered, in which the students explored natural selection in Peppered moths in one of two different ways: either by actually 'hunting' paper moths in a more student centric active manner or by completing a scaffolded written activity after watching and discussing a power point presentation in a more teacher centred approach.

The 'hunting' moths activity involved a differentiated practical investigation in which newspaper and white paper moths were 'hunted' by the students using forceps 'beaks' against different backgrounds followed by some questions to consolidate. This activity was used to introduce the idea of selective predation producing differential survival and reproduction leading to changes in trait frequency in the moth population in subsequent generations. The Peppered moth was chosen as a realist example that the students could relate to:

> *"They did love the moth hunting activity, I think it was a great example, because sometimes I find in scientific schemes and things, the animals aren't very child friendly. They get moths. They get birds. That's it. It's real for them."*

Teachers reported that the although the activity took longer than expected to complete due the additional organisation needed to select the groups and distribute the resources the practical was *worthwhile, as it demonstrated the concept of natural selection very well.*

> *"The children found this really interesting, really interesting. They loved doing the activity, it took a long time to do, so it took most of the afternoon. We did it as a whole class activity, I had mixed year groups together so the logistics took a bit of organising. It did take a long time to do but it ensued the less able and the younger ones were still able to fill in the sheets and participate."*

> *"It [the moths] took a lot longer to do than I thought it would as they found it difficult to understand the instructions. But I think that was me rather than the kids. I did it to two classes and I was much better at it the second time, obviously I got it then and was better at explaining what to do."*

> *"They saw the concept very clearly, I think it was very visual, which was really good and they, did it without trying, they were absolutely picking up the ones that, you would expect, the ones that really stood out against the background"*

Once the small groups had been organised the students were able to just get on with the practical allowing the teacher to act as a facilitator drawing out extra details and developing their understanding of new concepts.

"We did it as a whole class activity, altogether. I said, "OK, go" and everybody did their things. And we were then sort of able to discuss, what are you finding, what's happening in this group, what's happening in that group as I walked round the room, so we did take a long time to do it. They also got the idea that it was a simulation, and what a simulation was, and that was a completely new concept, completely new to them. They found, not just, not just proving camouflage helps, so the white ones obviously you can't see on the white paper, so they can survive better. They actually found that there were patterns in the size as well. So, my focus had been, OK, well we're going to understand by the end of this session that it's the camouflage that is the key factor in survival and therefore reproduction. But they came out with if they're smaller, they're harder to get, as well."

They also remarked on the practical nature of the activity forming a point of reference underpinning the understanding of the concept of natural selection:

"The one with the moths and forceps was brilliant as well, that was something that then we could keep referring back to, so when we were discussing other things, we said, "Oh do you remember?" So, I think the fact that they'd done it practically really helped kind of embed that understanding about that one."

The alternative Peppered moth activity involved a step by step power point presentation explaining the process of natural selection with an embedded an interactive white board class simulation, followed by a scaffolded differentiated story board written exercise. Although less teachers commented on this activity than the previous one the majority of those who did comment reported that their students enjoyed the activity and thought the differentiated scaffolded writing frame was a successful way of describing the process.

"The white board moth story, they loved that as well. It helped really visualise it, you know, seeing the different environments that the Peppered moths were in. Yes, I think they did, and they did some nice write-ups on that as well, so that was one of the really good lessons that we did actually."

"We put the frame up and then they used that frame then to write the second one. Through the use of the scaffold, were they all able to access that to a lesser or greater extent. Yeah, it definitely helped. I think they would have struggled. Well, quite a lot of them would have struggled if they didn't have that."

However, one teacher felt that this activity was not successful in their class.

"We found it quite complicated as well for them. They have to have very, very visual stuff or hands on things and that was a little bit, it just wasn't that interesting."

These mainly positive comments demonstrate that teachers endorsed the use of both different moth activities as a method of introducing natural selection in primary age students. However, using feedback

obtained from the small number of teachers who participated in the study over two consecutive years we can explore individual teacher preference by direct comparison of the two moth activities taught by the same teacher. Again, this feedback suggests that both activities were successful in demonstrating the principle of natural selection and engaging student interest and is consistent with the statistical testing carried out in the previous section. Additionally, the choice of which of the two activities they would use in the future would depend on the ability of their students, the time available and the availability of resources.

"I had the white board moth activity this year and that went really well, I think again it's a really, really brilliant way of showing how things survive, it worked well last time to be honest when we hunted the moths. I know it's the same concept but just done in a slightly different way. Both were really good and the seed has been Scheme of Work for them for their interest in the future, which I think is really important".

"Having done both [moth activities], I think my top set really enjoyed hunting the paper moths. I think with my parallel sets [middle ability] and low ability it was much easier to manage the white board one, because just organising their equipment and counting out things takes so much time that we were getting to the point of no return and losing things. I think it was it was much easier to use the computer with the write up sheet with my parallel groups".

If attention is now turned to the concept of common ancestry by studying homology in lesson 4, the data demonstrates that the teachers felt that the pentadactyl limb activity was an interesting way to introduce the concept of common ancestry through the study of homologous bones within the forelimbs of different mammals. Teachers in this study reported that their students enjoyed the activity and that it really helped them to visualise our links to other mammals and shared common ancestry.

"They really enjoyed doing that [limb activity] and seeing the bones and I think again the visual of actually making them was great."

"They liked that [limb activity]. I think they found it really interesting to see how, you know, how the bones had adapted and changed their positions all the way through from the sheet and colouring in. They liked that they could sort of see the horse's hoof and all the rest of it, and the salt dough, they liked the salt dough."

"We had the pentadactyl limbs and we made salt dough life-size versions of them. They got into little groups and they made full limbs and everything, which was really interesting. They were able to label all of them. Again, it was one of those where they saw it, and then they could see the adaptations and that reinforced the having one single ancestor and how that all worked through. That was really interesting, for them. It was quite cool for me as well. There were a few 'penny drop' moments throughout the room. Then they were like, oh yeah, it's the same."

"They found that [the limbs] really interesting actually looking at how all of these different animals, skeletons had the same bones in and identifying the bones in the different animals and trying to work out which animals they were. They learnt a lot in that lesson and could understand how we share the same common ancestor."

Teacher preconceptions over concerns that the activity seemed too complicated and difficult for the students to complete before teaching it were allayed:

"They had the limb. It was amazing actually. I thought they'd find it more difficult than they did. There was very few that needed me to go over and really point out what was going on. Yeah, they really picked up on it. When you look at that task, you think they're never going to be able to do that because it looks too complicated. But that's what I thought and they just sort of got their heads down and did it."

The teachers also felt that the Trilobite activity in lesson 4 was an interesting way to study how adaptations can aid an organism's survival in different habitats. They reported that the students enjoyed the practical activity and that it generated some valuable discussions furthering their understanding.

"They loved that one [Trilobites]. The salt dough, that was brilliant, and showing the adaptions. They had to explain their choices. They took the different sections and they had to show adaptions on those sections, then describe them and because of all that learning that went before, they could easily justify their adaptions and why they would possibly work. The range of discussions you'd get within a group were just amazing."

"Oh, yes, the salt dough trilobites. They very much enjoyed those. We had some very interesting looking things and quite a lot of flour all over the floor. They loved that. A nice practical thing. They were saying things like "Am I supposed to have that"? They look over and think, mine doesn't look like that. Yours won't because it lives somewhere else, and they're like, oh good. Actually, this type of talk and sharing their ideas helps them have a better understanding of their own knowledge."

"They enjoyed that [the Trilobite activity] At first, they were just loving the dough, it was a great activity. And then they had to talk about their species, how it was adapted, where it was living. Whether it would survive or not and why."

"I thought the Trilobites particularly were a massive success. They absolutely loved it, because I think it brought to life what's a very difficult subject, I mean obviously you can look at fossils and everything else. But I think making something of their own that looks like something from millions of years ago was just brilliant. The class was able to choose their own adaptions, and so they talked about their trilobite and where it would live and how it would survive and all of those sorts of things. I think it was very, very successful drawing things together at the end of the topic."

However, none of the teachers commented on the intended link to common ancestry through the study of homologous body plans in *Trilobite sp.* unlike the comments obtained from the pentadactyl limb activity.

Additionally, some of the teachers did not seem to particularly like this activity as it was perceived to be boring and out of context with the rest of the sequence of lessons:

"We got trilobites, and the kids said, "Oh, not more paper." The colouring in exercise really didn't work as they would have preferred to have fossils on the table, magnifying glasses and the chrome books and identified what those fossils were and looked to see what they related to now. So again, they wanted to be more hands on."

Interestingly, when repeating teachers were asked for their preference between the main activities for lesson 4 (either pentadactyl limb or Trilobites) opinions were mixed and depended on class interests and student ability.

"We enjoyed the trilobites more than the limb from last year. It's funny isn't it? I thought the bones would be more relevant to most children but this group were very interested in fossils, a lot of them brought in their own fossils to show. So, I think that's maybe why it appealed to them, I'm not really sure, but possibly that's why it appealed to them so much."

"We loved them [the Trilobites]. They really enjoyed it but it wasn't that different from the previous one [limb] which was also really good. I was a bit concerned about it [using this with my low ability group] but once we did one or two together they sort of flew a bit and actually they enjoyed doing it on their own, as I think they felt it was more challenging than [the work] they normally have but more manageable than limbs which were more complicated. They seemed quite chuffed, like we're doing this bit of work on our own and we can explain what we're doing."

Supplementary Note 8: All four variable activities were successful

The teachers felt that the choice of organisms was appropriate to illustrate the complex concepts being introduced

"*They did love the moth hunting activity, I think it was a great example, because sometimes I find in scientific schemes and things, the animals aren't very child friendly. They get moths. They get birds. That's it. It's real for them.*"

"*The white board moth story, they loved that as well. It helped really visualise it, you know, seeing the different environments that the peppered moths were in. Yes, I think they did, and they did some nice write-ups on that as well, so that was one of the really good lessons*"

"*They found that [the limbs] really interesting actually looking at how all of these different animals, skeletons had the same bones in and identifying the bones in the different animals and trying to work out which animals they were. They learnt a lot in that lesson and could understand how we share the same common ancestor.*"

"*I thought the Trilobites particularly were a massive success. They absolutely loved it, because I think it brought to life what's a very difficult subject, I mean obviously you can look at fossils and everything else. But I think making something of their own that looks like something from millions of years ago was just brilliant. The class was able to choose their own adaptions, and so they talked about their trilobite and where it would live and how it would survive and all of those sorts of things. I think it was very, very successful drawing things together at the end of the topic.*"

However, their opinion as to which activity they preferred or would use again were mixed and depended upon class interests, the ability of their students, the time available and the availability of resources. For example, if we consider the feedback obtained from teachers who participated over two consecutive years, we can explore individual teacher preference by direct comparison of the two moth activities to introduce the concept of natural selection in Lesson 2:

"*I had the white board moth activity this year and that went really well, I think again it's a really, really brilliant way of showing how things survive, it worked well last time to be honest when we hunted the moths. I know it's the same concept but just done in a slightly different way. Both were really good and the seed has been Scheme of Work for them for their interest in the future, which I think is really important*".

"*Having done both [moth activities], I think my top set really enjoyed hunting the paper moths. I think with my parallel sets [middle ability] and low ability it was much easier to manage the white board one, because just organising their equipment and*

counting out things takes so much time that we were getting to the point of no return and losing things. I think it was it was much easier to use the computer with the write up sheet with my parallel groups".

Similar comments were obtained when asked for their preference between the main activities for Lesson 4 (either pentadactyl limb or Trilobites):

"*We enjoyed the trilobites more than the limb from last year. It's funny isn't it? I thought the bones would be more relevant to most children but this group were very interested in fossils, a lot of them brought in their own fossils to show. So, I think that's maybe why it appealed to them, I'm not really sure, but possibly that's why it appealed to them so much."*

"*We loved them [the Trilobites]. They really enjoyed it but it wasn't that different from the previous one [limb] which was also really good. I was a bit concerned about it [using this with my low ability group] but once we did one or two together they sort of flew a bit and actually they enjoyed doing it on their own, as I think they felt it was more challenging than [the work] they normally have but more manageable than limbs which were more complicated. They seemed quite chuffed, like we're doing this bit of work on our own and we can explain what we're doing."*

However, none of the teachers commented on the intended link to common ancestry through the study of homologous body plans in *Trilobite sp.*, unlike the comments obtained from the pentadactyl limb activity:

"*We had the pentadactyl limbs and we made salt dough life-size versions of them. They got into little groups and they made full limbs and everything, which was really interesting. They were able to label all of them. Again, it was one of those where they saw it, and then they could see the adaptations and that reinforced the having one single ancestor and how that all worked through. That was really interesting, for them. It was quite cool for me as well. There were a few 'penny drop' moments throughout the room. Then they were like, oh yeah, it's the same.*"

Supplementary note 9 – Student questionnaire

Student Questionnaire

GEVO2each project

Directions – Personal information

- Fill in your personal details on the front of the answer sheet
- Make sure that you include your date of birth, including the year.
- The name of your science teacher is the teacher that normally teaches you for science.

How to do the test

- Your teacher is now going to read out the questions one by one
- There are 15 multiple choice questions
- Listen carefully to each question
- Circle ONE correct answer (A,B,C or D)
- Your teacher will give you time to think and will let you see the question on the board if you need it.
- You MUST circle an answer for all 15 questions.
- A trait is a characteristic e.g. Eye colour.

Question 1

Some organisms, like a human and a chimpanzee (chimp) have many similarities. Others like a lion and a worm have fewer. The circles show organisms that share a common ancestor.

Which answer is TRUE about who shares a common ancestor?

Select one answer from the options below.

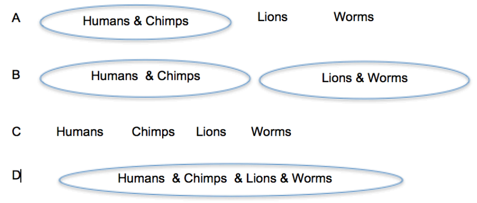

Question 2

Which answer is **TRUE** about a species living today and an extinct species?

(A) A living species and an extinct one **can share a common ancestor,** but only if they have **lots of similarities.**
(B) A living species and an extinct one **can share a common ancestor** that lived a long time ago. Even if they have **few similarities.**
(C) A living species **can't share** a common ancestor with an extinct one.
(D) **Only living species can share a common ancestor** with each other. Extinct species can't share a common ancestor with each other.

Question 3

This question is about the evolution of plants and animals. The circles show organisms that share a common ancestor.

Which answer is TRUE about who shares a common ancestor?

Select one answer from the options below.

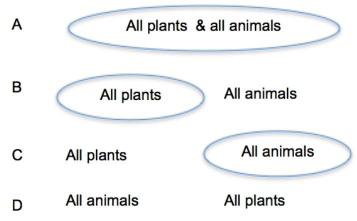

Question 4

What would happen to a species of lizards when a new predator appears where the lizards live?

(A) Lizards that **already have traits** that help them avoid the predator would be **more likely to survive and reproduce**. The ones that don't would be less likely to survive and reproduce.

(B) **All** lizards would **try to develop** new traits to avoid the new predator.

(C) **Some** lizards would **try to develop** new traits to avoid the new predator. The others would die.

(D) All the lizards have the **same traits** because they are from the **same species**. All of them have the **same chance** of surviving or dying.

Question 5

A species of bird had different sized beaks and ate many types of seeds.

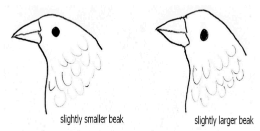

The climate changed to be much drier and only much larger seeds were left.

Which answer **best explains** why almost all of the birds had slighter larger beaks after many generations?

(A) The birds with **bigger beaks** could eat the big seeds. Only these birds got enough food to survive and **pass on the big beak trait** to their offspring.

(B) Birds with **smaller beaks worked** really hard to open the big seeds. Their beaks got **bigger** because they **used** them. They could then get enough food to survive and pass on the big beak trait.

(C) Birds with **smaller beaks grew** their beaks because they they **wanted** to open the seeds to get enough food to survive and pass on the big beak trait.

(D) It was **chance** that **all the birds' beaks got bigger** in one generation. They could then get enough food to survive and pass on the big beak trait.

Question 6

Could members of a species today look different to individuals of the same species from many generations ago?

(A) **YES**. **All** individuals can change a little in their **lifetime** and pass on those changes to their offspring.

(B) **YES. Some** individuals can change a little in their **lifetime** and pass on those changes to their offspring.

(C) **YES**. Some individuals **born** with certain traits are more likely to survive and pass on these traits to their offspring.

(D) **NO**. Species do not change even after many generations. So individuals of the same species wouldn't look different.

Question 7

Which of these is **NEEDED** for the process of natural selection to occur?

(A) Members of the **same species** must **compete** with each other.

(B) Members of **different species** must **compete** with each other.

(C) There must be a sudden **environmental change**.

(D) **Traits must be inherited** from one generation to the next

Question 8

Members of the same species can inherit different traits.

What could these differences change?

Select one answer from the table below.

Answer	Their ability to find food	Their ability to attract a mate
A	✔	✔
B	✔	✗
C	✗	✔
D	✗	✗

Question 9

Which answer is **TRUE** about members of the same species?

(A) They may have **inherited different traits**. These differences may **change their chance** of surviving and reproducing**.**

(B) They would **all have the same inherited traits** because they are from the **same species**. So they all have an **equal chance of** surviving and reproducing**.**

(C) They all have the **same inherited traits**. But they **pick up** different skills and learn new things during their **lifetime**. Only these **new skills change their chance** of surviving and reproducing.

(D) They may have **inherited different traits**. But these different traits **do not change their chance** of surviving and reproducing.

Question 10

Which answer **best describes** what a fossil can be?

Select one answer from the table below.

Answer	A bone in which the original matter has been replaced by rock	An impression left by a bone in rock
A	✔	✗
B	✗	✔
C	✔	✔
D	✗	✗

Question 11

Which answer is **TRUE** about extinction of species?

(A) **Very few species** have ever become **extinct**. Most are still alive.

(B) There have been **extinction events** when **many** species became **extinct** at about the **same time**. Apart from these extinction is very rare.

(C) **Humans** have caused the majority of extinctions. Up until recently species rarely became extinct.

(D) **Many species** have become **extinct throughout the history** of life on earth.

Question 12

A scientist finds some fossils of an extinct species of fish.

What can she do by studying the fossils?

Select one answer from the table below.

Answer	Discover what features the extinct species had	Discover similarities and differences between features of the extinct species and those alive today
A	✔	✔
B	✔	✗
C	✗	✔
D	✗	✗

Question 13

Which answer is **TRUE** about oak trees and lizards?

Select one answer from the table below.

Answer	There are similarities between oak trees and lizards	There are differences between oak trees and lizards
A	✓	✓
B	✓	✗
C	✗	✓
D	There is no way to tell	

Question 14

Which answer is **TRUE** about the species that are living on earth today?

(A) **All species living** today have **existed since the time life began.**

(B) **Most** species living today **have existed since the time life began**. But a **few** have appeared more **recently.**

(C) **Most** species alive today **didn't exist** at the time life began.

(D) There is **no way of finding out**. You can't tell whether all, most or a few species living today have existed since the time life began.

Question 15

Which answer is **TRUE** about how environmental conditions have changed since life began on earth?

(A) Conditions have remained about the **same everywhere** on earth. Only **minor changes** from year to year.

(B) Conditions have remained the **same in the oceans** but have **changed on land**.

(C) Conditions have remained **the same except** for a **few sudden changes** in **certain places**. Such as a meteorite hitting the earth.

(D) Conditions have **changed dramatically**. Some of these changes have happened suddenly and others more gradually.

Supplementary Note 10

Student questionnaire – written version

Student Questionnaire

Instructions:

- Please fill in the front of this booklet.
- This assessment has 2 sections.
- There are 15 multiple-choice questions and then a table about an adoption story to complete.
- Answer all of the questions in the booklet.
- Please do not work with anyone else or share answers
- Don't worry if you don't know an answer.

Your Name: ___________________

Your Date of Birth (including year)__________________

Are you a Boy or a Girl?____

Name of your School: ____________________________

Name of Science Teacher: ______________________

Date:___________________

Pre	Post	Retention

- Your answers will be kept strictly confidential during this study.
- Your answers will not affect your science grades at school.
- By completing this questionnaire you will be helping research by the Evolution Education Trust and the University of Bath.

Thank you for your help

THE EVOLUTION EDUCATION TRUST   UNIVERSITY OF BATH

For each question please circle ONE letter to show the correct answer. A trait = a characteristic e.g. eye colour.

1. Some organisms, like a human and a chimpanzee (chimp) have many similarities. Others like a lion and a worm have fewer. The circles show organisms that share a common ancestor.
   Which answer is TRUE about who shares a common ancestor? Select one answer from the options below.

A (Humans & Chimps) Lions Worms

B (Humans & Chimps) (Lions & Worms)

C Humans Chimps Lions Worms

D (Humans & Chimps & Lions & Worms)

2. Which answer is **TRUE** about a species living today and an extinct species?

   A. A living species and an extinct one **can share a common ancestor**, but only if they have **lots of similarities**.
   B. A living species and an extinct one **can share a common ancestor** that lived a long time ago. Even if they have **few similarities**.
   C. A living species **can't share** a common ancestor with an extinct one.
   D. **Only living** species **can share a common ancestor** with each other. Extinct species can't share a common ancestor with each other.

3. This question is about the evolution of plants and animals. The circles show organisms that share a common ancestor.

Which answer is TRUE about who shares a common ancestor?

Select one answer from the options below.

A (All plants & all animals)

B (All plants) All animals

C All plants (All animals)

D All animals All plants

4. What would happen to a species of lizards when a new predator appears where the lizards live?

A. Lizards that **already have traits** that help them avoid the predator would be **more likely to survive and reproduce**. The ones that don't would be less likely to survive and reproduce.
B. **All** lizards would **try to develop** new traits to avoid the new predator.
C. **Some** lizards would **try to develop** new traits to avoid the new predator. The others would die.
D. All the lizards have the **same traits** because they are from the **same species**. All of them have the **same chance** of surviving or dying.

5. A species of bird had different sized beaks and ate many types of seeds

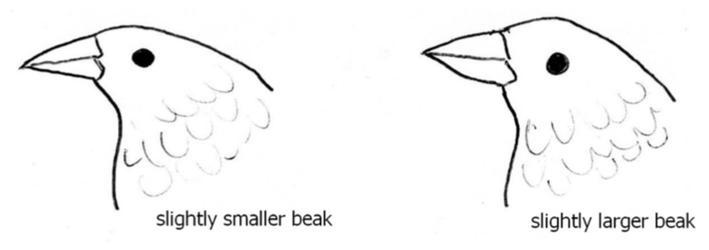

The climate changed to be much drier and only much larger seeds were left.

Which answer best explains why almost all of the birds had slighter larger beaks after many generations?

A. The birds with **bigger beaks** could eat the big seeds. Only these birds got enough food to survive and **pass on the big beak trait** to their offspring
B. Birds with **smaller beaks worked** really hard to open the big seeds. Their beaks got **bigger** because they **used** them. They could then get enough food to survive and pass on the big beak trait.
C. Birds with **smaller beaks grew** their beaks because they they **wanted** to open the seeds to get enough food to survive and pass on the big beak trait.
D. It was **chance** that **all the birds' beaks got bigger** in one generation. They could then get enough food to survive and pass on the big beak trait.

6. Could members of a species today look different to individuals of the same species from many generations ago?

A. **YES**. **All** individuals can change a little in their **lifetime** and pass on those changes to their offspring.
B. **YES. Some** individuals can change a little in their **lifetime** and pass on those changes to their offspring.
C. **YES**. Some individuals **born** with certain traits are more likely to survive and pass on these traits to their offspring.
D. **NO**. Species do not change even after many generations. So individuals of the same species wouldn't look different.

7. Which of these is **NEEDED** for the process of natural selection to occur?

A. Members of the **same species** must **compete** with each other.
B. Members of **different species** must **compete** with each other.
C. There must be a **sudden environmental change**.
D. **Traits must be inherited** from one generation to the next

8. Members of the same species can inherit different traits.
What could these differences change?
Select one answer from the table below.

Answer	Their ability to find food	Their ability to attract a mate
A	✔	✔
B	✔	✗
C	✗	✔
D	✗	✗

9. Which answer is **TRUE** about members of the same species?

A. They may have **inherited different traits**. These differences may **change their chance** of surviving and reproducing.

B. They would **all have the same inherited traits** because they are from the **same species**. So they all have an **equal chance** of surviving and reproducing.
C. They all have the **same inherited traits**. But they **pick up** different skills and learn new things during their **lifetime**. Only these **new skills change their chance** of surviving and reproducing
D. They may have **inherited different traits**. But these different traits **do not change their chance** of surviving and reproducing.

10. Which answer best describes what a fossil can be?
Select one answer form the table below.

Answer	A bone in which the original matter has been replaced by rock	An impression left by a bone in rock
A	✔	✗
B	✗	✔
C	✔	✔
D	✗	✗

11. Which answer is **TRUE** about extinction of species?

A. **Very few species** have ever become **extinct.** Most are still alive.
B. There have been **extinction events** when **many** species became **extinct** at about the **same time**. Apart from these extinction is very rare.
C. **Humans** have caused most of the extinctions. Up until recently species rarely became extinct.
D. **Many species** have become **extinct throughout the history** of life on earth.

12. A scientist finds some fossils of an extinct species of fish.
What can she do by studying the fossils?
Select one answer from the table below.

Answer	Discover what features the extinct species had	Discover similarities and differences between features

		of the extinct species and those alive today
A	✔	✔
B	✔	✗
C	✗	✔
D	✗	✗

13. Which answer is **TRUE** about oak trees and lizards?
Select one answer from the table below.

Answer	There are similarities between oak trees and lizards	There are differences between oak trees and lizards
A	✓	✓
B	✓	✗
C	✗	✓
D	There is no way to tell	

14. Which answer is **TRUE** about the species that are living on earth today?

A. **All species** living today have **existed since the time life began**.
B. **Most** species living today **have existed since the time life began**. But a **few** have appeared more **recently.**
C. **Most** species alive today **didn't exist** at the time life began.
D. There is **no way of finding out**. You can't tell whether all, most or a few species living today have existed since the time life began.

15. Which answer is **TRUE** about how environmental conditions have changed since life began on earth?

A. Conditions have remained about the **same everywhere** on earth. With only **minor changes** from year to year.
B. Conditions have remained the **same in the oceans** but have **changed on land.**
C. Conditions have remained the **same except** for a **few sudden changes** in **certain places**. Such as a meteorite hitting the earth.
D. Conditions have **changed dramatically**. Some of these changes have happened suddenly and others more gradually.

Select one answer from the table below.

Answer	Discover what features the extinct species had	Discover similarities and differences between features of the extinct species and those alive today
A	✔	✔
B	✔	✗
C	✗	✔
D	✗	✗

13. Which answer is **TRUE** about oak trees and lizards?
Select one answer from the table below.

Answer	There are similarities between oak trees and lizards	There are differences between oak trees and lizards
A	✓	✓
B	✓	✗
C	✗	✓

D	There is no way to tell

14. Which answer is **TRUE** about the species that are living on earth today?

A. **All species** living today have **existed since the time life began**.
B. **Most** species living today **have existed since the time life began**. But a **few** have appeared more **recently.**
C. **Most** species alive today **didn't exist** at the time life began.
D. There is **no way of finding out**. You can't tell whether all, most or a few species living today have existed since the time life began.

15. Which answer is **TRUE** about how environmental conditions have changed since life began on earth?

A. Conditions have remained about the **same everywhere** on earth. With only **minor changes** from year to year.
B. Conditions have remained the **same in the oceans** but have **changed on land.**
C. Conditions have remained the **same except** for a **few sudden changes** in **certain places**. Such as a meteorite hitting the earth.
D. Conditions have **changed dramatically**. Some of these changes have happened suddenly and others more gradually.

Supplementary Note 11: Misinterpretation of assessment items

Assessment items 1 and 3 probed the understanding of common ancestry by giving different combinations of organisms that could potentially share a common ancestor. In item 1 the organisms given were specific named examples: humans, chimps, worms and lions, whilst in item 3 the options were more general and restricted to the terms all animals and plants. Interestingly these contextual differences elicited very different responses from the students.

Despite the focus groups being conducted after the teaching of the whole Inheritance and selection topic ¾ of the focus groups failed to give the correct answer for question 1 opting for only chimps and humans sharing common ancestry.

G1 *"Because they're like closer together than lions and worms. They have more in common. And we were chimps...And we've evolved into humans from chimps."*

G3 *"It's because we've evolved from chimps. We are more similar to chimps so we must be related to them."*

G4 "*Yeah, humans and chimps share a common ancestor, but lions and worms don't."*

However, ¾ of groups gave the correct answer to question 3, that all animals and plants share a common ancestor.

G2 *"All of them share a common ancestor. We've all evolved from a common ancestor. The first single-celled organism."*

When probed as to the reason for this discrepancy the following explanations were given:

G1 *"we didn't give the right answer before [in question 1] but did in this one [question 3] 'cause they never said about lions and worms. And worms are really like tiny and they don't have any arms and legs like humans and chimps, and lions are like on four legs, and they're different. it's because we know what lions and worms look like."*

G2 *"I can see why they might give the wrong answer. Because like, we know that our nearest ancestor is a chimp, so then they'd go for that. And it also says, 'Others, like lion and worm, have fewer,' so that makes me think that they might not go for these."*

G4 *"Cause we wouldn't think that, like… Worms and lions would have less in common…you don't think humans can share a relative with worms."*

G1 *"I knew like, we were like the same as like animals, but like flowers, like I didn't really understand, that technically, like they all breathe and... so they're the same. Yeah, 'cause we all breathe, so we're all the same."]*

We can go onto consider these comments in more detail by analysing each assessment item by option chosen by the whole cohort of students. Assessment item 3 considered the shared common ancestry of all plants and animals in general terms. Response A was the correct answer in which all plants and animals share a common ancestor, with responses B and C both representing the alternative conception that animals and plants do not share a common ancestor (Bizzo, 1994) and D representing the alternative conception that members of different species do not share a common ancestor (Poling & Evans, 2004b; Shtulman, 2006). Pre-instruction option C that only animals share a common ancestor with each other was the was the most frequent response (28.83%) followed by the correct response A (26.69), interestingly option B in which only plants share a common ancestor was less frequent (21.83%). Post instruction this item showed the most significant overall difference in test values ($F = 33.07$, $P = 3.9\text{x}10^{-11}$ repeated measures one-way ANOVA; see Supplementary Table 7). There was a highly significant conversion to the correct response from all distractors in both the post ($P = 3.3\text{x}10^{-8}$) and retention ($P = 4.2\text{x}10^{-7}$) tests, the correct response (A) becoming the highest scoring answer in both. There was some degree of waning from the correct response in the post-test to the retention test, however this was not a significant ($P = 0.06$). The results of this assessment item suggest that instruction was successful in both improving and retaining the correct understanding of this concept. Additionally, there was a sequential decrease in the percentage of students opting for response D suggesting instruction was particularly successful in addressing the alternative conception that members of different species do not share a common ancestor.

Assessment item 1 proved to be the most challenging assessment item for the students, achieving the lowest score on all three tests. The correct response was D in which chimpanzees, humans, zebras, and worms all share a common ancestor, with responses A and C acting as attractive distractors. Response A was based on the common alternative conception that only similar species (humans and chimpanzees) can share a common ancestor, whilst (lions and worms) species with no obvious similarities cannot (Hagay, 2005; Poling & Evans, 2004b; Shtulman, 2006), whilst response C was based another common alternative conception that members of different species do not share a common ancestor (Poling & Evans, 2004b; Shtulman, 2006). This item showed a highly significant overall difference in test values post instruction ($\chi^2 = 33.99$, $P = 4.17\text{x}10^{-8}$, Friedman test; see Supplementary Table 8). Pre-instruction option A was by far the most frequent response (84.70%) showing a strong preference for the alternative conception of only closely related species sharing a common ancestor, whilst the correct option frequency was very low (4.39%). Post instruction there was a highly significant conversion to the correct response from all distractors in both the post ($P = 1.9\text{x}10^{-6}$, post hoc Friedman Nemenyi test) and retention ($P = 6.4\text{x}10^{-5}$, post hoc Friedman Nemenyi test) tests, although response A remained the highest scoring answer. There was some degree of waning from the correct response in the retention back to response A, however this was not a significant

(P = 0.75, post hoc Friedman Nemenyi test) suggesting that instruction was successful in correcting preconceptions in some students to the accepted scientific understanding in the longer term. However, it must be noted that the alternative conception represented by response A was both prevalent and persistent remaining as the highest scoring response in all tests, suggesting a deeply held belief that humans and chimpanzees can share a common ancestor as they are so similar. This finding seemingly contradicts the results from assessment item 3 in which students show a much better understanding that all animals and plants share a common ancestor and could be result of the human related context of the assessment item reinforcing this alternative conception.

Supplementary Note 12: Plain English statement

UNIVERSITY OF BATH

The Evolution Education Trust

Plain Language Statement for students

My name is Dana Buchan. I am a first year PhD student in Biology and Biochemistry at the University of Bath. I would like to invite you to participate in my research project entitled *"What is the most effective way of teaching evolution to primary school children?"* This project is under the supervision of Professor L Hurst

Invitation paragraph

You are being invited to take part in a research study. Before you decide it is important for you to understand why the research is being done and what it will involve. Please take time to read the following information carefully and discuss it with others if you wish. Please ask if there is anything that is not clear or if you would like more information. Take time to decide whether or not you wish to take part.

What are the purposes of the study?

This study aims to investigate (a) The development and assessment of the best scheme of work and teaching resources for the year 6 NC evolution topic (b) Assessment of pupil understanding of evolutionary biology and its evidence, (c) Pupil learning approaches to evolution, and (d) the assessment of the influence of teacher understanding and acceptance of evolution.

Why have I been chosen?

You are being approached because you are a year 6 pupil in a participating primary, junior or middle school.

Do I have to take part?

No. Your participation is voluntary. Although you decide to take part in this research, you are able to withdraw your participation at any time.

What will happen to me if I take part?

If you agree to take part in this research, you will be taught the evolution topic using resources that I have written. You will be given a questionnaire to assess your understanding of natural selection and evolution, before you are

taught the topic, just after it and possible 6 months afterwards. Each questionnaire will take approximately 30 minutes to complete.

Will my taking part in this study be kept confidential?

Yes, of course. You will put your names on the questionnaires but when I analyse them I will give you a special code to identify you. Your name will not appear in my study. In addition, the returned questionnaires will be kept in locked filing cabinet at the University of Bath. At the end of my study, all of returned questionnaires will be shredded.

What will happen to the results of the research study?

The results will be analysed by statistical tools as well as qualitatively. The research data will be typed up as a PhD dissertation, journal articles, conference papers and other academic purposes, which you can access (if required).

Who is organising and funding the research?

This research is organised by me, Dana Buchan, through the University of Bath, funded by the Evolution Education Trust. It is under the supervision of Professor L Hurst, the main supervisor and Dr M Hejmadi my second supervisor.

Who has reviewed the study?

Apart from the names of the people mentioned above, this research has been reviewed and approved by ethics committees of the University of Bath.

Contact for Further Information

If you have any other questions about the research project, you can contact me (Mrs Dana Buchan) directly by phone on (+44) 07549947874 or by email on edmllb@bath.ac.uk.

Supplementary Note 13: Teacher consent form

GEVO2teach Teacher Focus Group Information Sheet

This focus group is part of my PhD project that is looking at how best to teach Evolution to year 6 students.

- This focus group discussion should last between 45-60 minutes.
- The focus group will be recorded, and then transcribed so that I have a record of the discussion.
- When the focus group's discussion is transcribed I will change your name and any references that you make to specific people and places to ensure that no-one can be identified from our data.
- Nothing you say in this focus group will be heard by anyone else in your school or at the University. All responses are confidential and I ask that all focus group participants respect the privacy and views of other group members.
- If you agree to take part in this focus group, but feel at any stage that you would like to stop, you are free to do so at any time, without giving any reason.

If you have any questions about this project, then feel free to contact me:

Dana Buchan
GEVO2teach Project Postgraduate Researcher
Department of Biology and Biochemistry
University of Bath
BA2 7AY
tel: 01225 385902
email: l.buchan@bath.ac.uk

(Participant keeps this section)

Teacher Consent Form

Statement of consent:

I have read and understood the information sheet for the GEVO2teach research project and I have had the opportunity to ask any questions I have about the research.

I agree to participate in a focus group for the GEVO2tech project that will be audio-recorded and transcribed. Transcripts will be used only for the purposes of this research project, for as long as this research is being undertaken.

The researchers will not use my personal data for any other purpose or disclose it to any third parties. The information I provide, in the form of my comments, will be anonymised (e.g. my name will be removed and replaced with a number). I agree to parts of what I say being used anomalously by the researchers in publications and presentations.

Participant Name ____

Participant Signature ____

Researcher Name ____

Researcher Signature ____

Date ____

(Two copies required: one to be kept by the interviewee, one to be kept by the researcher)

~ Thank you ~

Supplementary Note 14

Student focus group consent form

Date

Dear Parent/Guardian,

I am pleased to inform you that your son/daughter has been invited to participate in a focus group for the University of Bath's GEVO2teach Primary Evolution Project. Please read this information sheet and complete the attached permission form if you are happy for your son/daughter to participate.

What the project is about

The GEVO2teach Project is an exciting new research initiative that aims to improve the teaching of evolution in primary schools. This study is under the supervision of Professor Laurence D Hurst from the Department of Biology and Biochemistry at the University of Bath. This research is funded by the Evolution Education Trust.

What the focus group is about

The purpose of this focus group is to explore young peoples' views and knowledge of natural selection and evolution. These are topics that your son/daughter has recently studied as part of their new Key Stage 2 National Curriculum using resources developed by this project.

What your son/daughter will be asked to do

Your son/daughter will be asked to participate in a focus group. This will take the form of a relatively informal small discussion group with approximately five other pupils from their science class. Each pupil will be asked opinion-based questions related to what they have learnt in their science classes. They will also be invited to respond to comments other members of the group make. The session will last no longer than one hour and will take place during your son/daughter's normal science lesson, in their school, on a date specified by their teacher. A member of school staff will remain within sight of the group for the duration of the session.

Benefits and risks

Findings from this research may help improve how evolution is taught in primary schools, which could benefit current and future school pupils. This study may prove an interesting experience for pupils to gain insight into biology and education research and will also be an opportunity for pupils to reflect on their learning. No risks greater than those experienced in ordinary conversation are anticipated. Everyone involved in the focus group will be asked to respect the privacy of the other group members.

PTO

Taking part is entirely voluntary

Your decision as to whether or not your son/daughter can participate in this focus group will have no impact on their school education or on any current or future relationship with the University of Bath. Your son/daughter will be free to withdraw from the study at any time.

All responses are confidential

The focus group will be recorded using a dictaphone so that it can be transcribed later; no one else will hear this and the recording will be destroyed once transcribed. Your son/daughter will never be identified by name in the transcription of the focus group or in any reports published as part of this research. All data will be kept strictly confidential: all materials will be stored in a secure location The data collected will only be seen by members of the GEVO2teach research team.

If you have any queries, please speak to your son/daughter's science/class teacher or contact me directly by phone or email.

If you are **willing** for your son/daughter to be involved in this research, please complete the attached form and return it to their science/class teacher.

I thank you in advance for your support.

Yours faithfully,

Dana Buchan

Dana Buchan

GEVO2teach Primary Project Postgraduate Researcher

Department of Biology and Biochemistry

University of Bath

BA2 7AY

tel: 01225 385902

email: l.buchan@bath.ac.uk

Focus Group Permission Form

Statement of consent:

I have read and understood the attached information.

By signing below, I give permission for my son/daughter to participate in a focus group for the GEVO2teach Primary Project.

Pupil Name ____

Science/Class Teacher ____

Parent/Guardian Name ____

Signature ____

Date ____

~ Thank you ~

Supplementary Table 1: Summary of existing studies

Authors of paper	Sample size and context	Research method	Topic studied
**(Kargbo et al., 1980)**	32 Canadian children	Clinical interviews but no hypothesis tested	Acquisition of inherited traits
**(Springer, 1992)**	72 American children aged between 4 and 7	Structured oral interviews using cue cards	Ability to distinguish between biological and non-biological relationships
**(Springer, 1995)**	54 American children aged between 3 and 5	Structured oral interviews	Understanding that babies grow inside the mother and may inherit properties form her
**(Springer, 1996)**	121 American children aged between 4 and 7	Structured oral interviews	Resemblance of family members and that shared properties don't always entail kinship
**(Samarapungavan & Wiers, 1997)**	35 Dutch children aged between 8 and 12.	Oral interview based on semi structured questionnaire	Speciation
**(Solomon & Johnson, 2000)**	64 American children aged between 5-6.	Structured oral interviews	Understanding of biological inheritance
**(Evans, 2001)**	102 American children aged between 5-13. (Christian fundamentalist and non-fundamentalist.)	Structured interviews and parental questionnaires	Origins of evolutionist/antievolutionist beliefs
**(Solomon, 2002)**	28 American preschool children aged between 4 and 5	Oral interviews using case studies and visual prompts	Resemblance of family members based on race and apparel
**(Poling & Evans, 2004)**	68 American children aged between 4-9	Structured oral interview using photographs	Concepts of death and extinction
**(Venville et al., 2005)**	90 Australian children aged between 9 and 15	Interviews with qualitative data collection, followed by quantitative scoring	Understanding of basic inheritance and molecular genetics concepts involved in the theory of kinship
**(Venville & Donovan, 2007)**	17 Australian 6-7 year - old children	Oral interviews and analysis of reflective journals	Development of students' theory of biology with concepts of the gene and DNA based on intervention lessons
**(Chanet & Lusignan, 2008)**	French children aged 4-11	Analysis of lesson products	Designed 5 activities to introduce evolution and enhance scientific reasoning skills
**(Shtulman & Schulz, 2008)**	43 American children aged between 4-9	Interviews structured around picture book	Identification of intraspecific variation
**(Berti et al., 2010)**	39 Italian children aged between 7 and 9	Structured oral interviews	Conceptions about the origin of species
**(Browning & Hohenstein, 2013)**	62 English children aged between 5 and 8	Semi-structured interview oral interview	Use of narrative to promote understanding of evolution in primary school children
**(Legare et al., 2013)**	88 American children aged between 5-11	Structured interviews on narrative recall of stories	Influence of language used to explain evolutionary concepts on understanding evolutional change
**(Kelemen et al., 2014)**	61 American children aged between 5 and 8	Structured oral interviews consisting	Explanation of natural selection through story book intervention

		of open and closed questions	
**(Järnefelt et al., 2015)**	40 American children aged between 5-8	Interviews based on forced choice questions on picture cue cards about fictional species.	Essentialism and intraspecific variation
**(Shtulman et al., 2016**)	96 American children aged 4-12	Tutorials and interviews based around a picture book resource	Evolutionary explanations for biological adaptation

Supplementary Table 2: Table of P values from the correlation between LOESS residual scores and teacher attribute.

Table of P values from the correlation between LOESS residual scores and teacher attribute. Note: Significant results highlighted in green. To be significant Holm multi-test adjusted P value based on individual P values obtained from both tranches ≤ 0.05.

Teacher attribute	Tranche 1	Tranche 2	Fisher's combined P-value	Holm adjusted P-value
Increase in confidence	ϱ = 0.38, $P = 3.4 \times 10^{-2}$ Spearman's rank test	ϱ = 0.52, $P = 2.00 \times 10^{-3}$ Spearman's rank test	7.21×10^{-4}	7.7×10^{-3}
Years of experience	ϱ = -0.06, P = 0.72 Spearman's rank test	ϱ = 0.43, $P = 1.57 \times 10^{-3}$ Spearman's rank test	8.75×10^{-3}	0.09
Completion bias	W = 138, P = 0.18 Wilcoxon rank-sum test	W = 175, $P = 2.5 \times 10^{-2}$ Wilcoxon rank-sum test	0.03	0.27
Formal evolution education	W = 106, P = 0.20 Wilcoxon rank-sum test	W = 245, P = 0.10 Wilcoxon rank-sum test	0.10	0.74
Teacher gender	W = 218, P = 0.23 Wilcoxon rank-sum test	W = 235, P = 0.09 Wilcoxon rank-sum test	0.10	0.74
Highest qualification in Biology	χ^2 = 5.08, P = 0.28 Kruskal-Wallis rank-sum test	χ^2 = 6.02, P = 0.11 Kruskal-Wallis rank-sum test	0.14	0.83
Acceptance of evolution	ϱ = 0.27, P = 0.12 Spearman's rank test	ϱ = -0.07, P = 0.64 Spearman's rank test	0.28	1.00
Religious affiliation	χ^2 = 1.68, P = 0.43 Kruskal-Wallis rank-sum test	χ^2 = 2.81, P = 0.25 Kruskal-Wallis rank-sum test	0.34	1.00
Multiple participation	W = 105, P = 0.45 Wilcoxon rank-sum test	W = 209, P = 0.49 Wilcoxon rank-sum test	0.55	1.00

Number of students in the class	ϱ = -0.15, P = 0.37 Spearman's rank test	ϱ = 0.06, P = 0.68 Spearman's rank test	0.60	1.00
Understanding of natural selection	ϱ = 0.02, P = 0.91 Spearman's rank test	ϱ = 0.02, P = 0.88 Spearman's rank test	0.98	1.00

Supplementary Table 3 : Table of P values from the correlation between LOESS residual scores and school attribute. Note: Significant results highlighted in green. To be significant Holm multi-test adjusted P value based on individual P values obtained from both tranches ≤ 0.05.

School attribute	Tranche 1	Tranche 2	Combined P (Fisher's test)	Holm adjusted P value
Type (primary or middle)	W = 49, P = 0.24, Wilcoxon rank-sum test	W = 83, P = 0.02, Wilcoxon rank-sum test	P = 0.03	0.40
% FSM (free school meals)	ϱ = -0.47, P = 0.06, Spearman's rank correlation	ϱ = -0.20, P = 0.30 Spearman's rank correlation	P = 0.09	1.00
% of Special Educational Needs students (SEN)	ϱ = 0.41, P = 0.10, Spearman's rank correlation	ϱ = 0.20, P = 0.31, Spearman's rank correlation	P = 0.14	1.00
Religious affiliation	W = 24, P = 0.28, Wilcoxon rank-sum test	χ^2 = 1.49, P = 0.47, Kruskal-Wallis rank-sum test	P = 0.40	1.00
Category (academy, managed, independent)	χ^2 = 1.6, P = 0.45, Kruskal-Wallis rank-sum test	χ^2 = 2.3, P = 0.32, Kruskal-Wallis rank-sum test	P = 0.42	1.00
LEI (Living Environment Deprivation Index)	ϱ = 0.39, P = 0.13, Spearman's rank correlation	ϱ = 0.13, P = 0.51, Spearman's rank correlation	P = 0.24	1.00
% of students meeting the standard level expected at the end of Key Stage 2 (MSE)	ϱ = 0.15, P = 0.58, Spearman's rank correlation	ϱ = 0.11, P = 0.59, Spearman's rank correlation	P = 0.71	1.00
Teacher pupil ratio	ϱ = 0.26, P = 0.32, Spearman's rank correlation	ϱ = 0.09, P = 0.65, Spearman's rank correlation	P = 0.54	1.00
IDACI (Income Deprivation Affecting Children Index)	ϱ = 0.14, P = 0.60, Spearman's rank correlation	ϱ = 0.12, P = 0.55, Spearman's rank correlation	P = 0.70	1.00

Ofsted rating grade	W = 11, P = 0.62, Wilcoxon rank-sum test	χ^2 = 0.10, P = 0.95, Kruskal-Wallis rank-sum test	P = 0.90	1.00
ESI (Education, Skills and Training Deprivation Index)	ϱ = -0.10, P = 0.94, Spearman's rank correlation	ϱ = -0.03, P = 0.99 Spearman's rank correlation	P = 0.99	1.00
Index of Multiple Deprivation (IMD)	ϱ = 0.17, P = 0.51, Spearman's rank correlation	ϱ = 0.04, P = 0.82, Spearman's rank correlation	P = 0.78	1.00
% of students with English as an Additional Language (EAL).	ϱ = 0.08, P = 0.77, Spearman's rank correlation	ϱ = $4.10 \times 10^{-3,}$ P = 0.98, Spearman's rank correlation	P = 0.97	1.00
Size (Number on roll)	ϱ = -0.03, P = 0.93, Spearman's rank correlation	ϱ = -0.33, P = 0.09, Spearman's rank correlation	P = 0.98	1.00

Supplementary Table 4: summary of baseline demographic and pre-test scores for students in both tranches. Note: data taken from students completing both the pre and post-tests, maximum test score = 15.

Student attribute	Tranche	
	1 (N = 988)	2 (N = 1309)
Mean age (days)	4009 ± 1733.97	3902 ± 198.64
Gender break down	♂ 51.11%<br>♀ 48.89%	♂ 49.66%<br>♀ 50.34%
Mean pre-test score	5.51 ± 2.15	5.77 ± 2.25
Min pre-test score	0	0
Max pre-test score	13	14

Supplementary Table 5: Overview of the allocation of mean LOESS residuals to calculate interaction effects

	**Lesson 2**		
**Lesson 4**	**Hunting Moths (HM)**	**Paper Moths (PM)**	**Lesson 4 means**
**Trilobites (T)**	SoW 1	SoW 3	T mean
**Pentadactyl Limb (PL)**	SoW 2	SoW 4	PL mean
**Lesson 2 means**	HM mean	PM mean	Grand mean

Supplementary Table 6

CONSORT 2010 checklist of information to include when reporting a randomised trial*

Section/Topic	Item No	Checklist item	Reported on page No
**Title and abstract**			
	1a	Identification as a randomised trial in the title	yes
	1b	Structured summary of trial design, methods, results, and conclusions (for specific guidance see CONSORT for abstracts)	2
**Introduction**			
Background and objectives	2a	Scientific background and explanation of rationale	3-7
	2b	Specific objectives or hypotheses	29
**Methods**			
Trial design	3a	Description of trial design (such as parallel, factorial) including allocation ratio	6,7
	3b	Important changes to methods after trial commencement (such as eligibility criteria), with reasons	Methods p23
Participants	4a	Eligibility criteria for participants	Methods, p34
	4b	Settings and locations where the data were collected	Methods, p34
Interventions	5	The interventions for each group with sufficient details to allow replication, including how and when they were actually administered	Methods, p24-33
Outcomes	6a	Completely defined pre-specified primary and secondary outcome measures, including how and when they were assessed	Methods 29-30
	6b	Any changes to trial outcomes after the trial commenced, with reasons	n/a
Sample size	7a	How sample size was determined	See section consort declarations p
	7b	When applicable, explanation of any interim analyses and stopping guidelines	n/a
Randomisation:			
Sequence generation	8a	Method used to generate the random allocation sequence	Methods, p34-35
	8b	Type of randomisation; details of any restriction (such as blocking and block size)	Methods,

			p34-35
Allocation concealment mechanism	9	Mechanism used to implement the random allocation sequence (such as sequentially numbered containers), describing any steps taken to conceal the sequence until interventions were assigned	Methods, p34-35
Implementation	10	Who generated the random allocation sequence, who enrolled participants, and who assigned participants to interventions	Methods, p34-35
Blinding	11a	If done, who was blinded after assignment to interventions (for example, participants, care providers, those assessing outcomes) and how	See section consort declarations
	11b	If relevant, description of the similarity of interventions	Table 1
Statistical methods	12a	Statistical methods used to compare groups for primary and secondary outcomes	Results
	12b	Methods for additional analyses, such as subgroup analyses and adjusted analyses	Results
**Results**			
Participant flow (a diagram is strongly recommended)	13a	For each group, the numbers of participants who were randomly assigned, received intended treatment, and were analysed for the primary outcome	Tables 11 & 12
	13b	For each group, losses and exclusions after randomisation, together with reasons	Results
Recruitment	14a	Dates defining the periods of recruitment and follow-up	Methods
	14b	Why the trial ended or was stopped	See section consort declarations
Baseline data	15	A table showing baseline demographic and clinical characteristics for each group	Supplementary Table 4
Numbers analysed	16	For each group, number of participants (denominator) included in each analysis and whether the analysis was by original assigned groups	Table 12
Outcomes and estimation	17a	For each primary and secondary outcome, results for each group, and the estimated effect size and its precision (such as 95% confidence interval)	Results
	17b	For binary outcomes, presentation of both absolute and relative effect sizes is recommended	n/a
Ancillary analyses	18	Results of any other analyses performed, including subgroup analyses and adjusted analyses, distinguishing pre-specified from exploratory	Results
Harms	19	All important harms or unintended effects in each group (for specific guidance see CONSORT for harms)	See section consort declarations
**Discussion**			

Limitations	20	Trial limitations, addressing sources of potential bias, imprecision, and, if relevant, multiplicity of analyses	Discussion
Generalisability	21	Generalisability (external validity, applicability) of the trial findings	Discussion
Interpretation	22	Interpretation consistent with results, balancing benefits and harms, and considering other relevant evidence	Discusison
**Other information**			
Registration	23	Registration number and name of trial registry	See section consort declarations
Protocol	24	Where the full trial protocol can be accessed, if available	See section consort declarations
Funding	25	Sources of funding and other support (such as supply of drugs), role of funders	See section consort declarations

*We strongly recommend reading this statement in conjunction with the CONSORT 2010 Explanation and Elaboration for important clarifications on all the items. If relevant, we also recommend reading CONSORT extensions for cluster randomised trials, non-inferiority and equivalence trials, non-pharmacological treatments, herbal interventions, and pragmatic trials. Additional extensions are forthcoming: for those and for up to date references relevant to this checklist, see www.consort-statement.org.

Supplementary Table 7: Analysis of assessment item 3. Data shown as mean percentage. Notes: green highlighted row showing correct response, orange rows showing attractive distractors. **** = P ≤ 0.0001

Item 3 Response	Pre-test %	Post-test %	Δ post-pre %	Retention test %	Δ ret-pre %	Δ post-ret %
A	26.69	55.28	28.59****	46.26	19.57****	9.02
B	21.83	14.71	-7.12	19.45	-2.37	-4.74
C	28.83	20.17	-8.66	25.39	-3.44	-5.22
D	19.93	9.73	-10.20	8.54	-11.39	1.19
U	0.83	0.12	-0.71	0.36	-0.47	-0.24
NA	1.90	0.00	-1.90	0.00	-1.90	0.00

Supplementary Table 8. Analysis of assessment item 1. Data shown as mean percentage. Note: green highlighted row showing correct response, orange rows showing attractive distractors. **** = P ≤ 0.0001 using Freidman test: post hoc Friedman Nemenyi test.

Item 1 Response	Pre-test %	Post-test %	Δ post-pre %	Retention %	Δ ret-pre %	Δ post-ret %
A	84.70	60.97	-23.72	66.79	-17.91	-5.81
B	6.76	5.58	-1.19	4.98	-1.78	0.59
C	2.61	1.90	-0.71	0.71	-1.90	1.19
D	4.39	31.44	27.05****	27.40	23.01****	4.03
U	0.59	0.12	-0.47	0.12	-0.47	0.00
NA	0.95	0.00	-0.95	0.00	-0.95	0.00

Supplementary Figure 1. Density plots showing correlation between matched pre and post-test scores. The denser the point, the more frequently it occurs. Note**;** tranche 1: N = 988, mean pre-test score = 5.51 ± 2.15, mean post-test score =7.92 ± 2.38; tranche 2, N = 1309, mean pre-test score = 5.77 ± 2.25, mean post-test score = 8.10 ± 2.70.

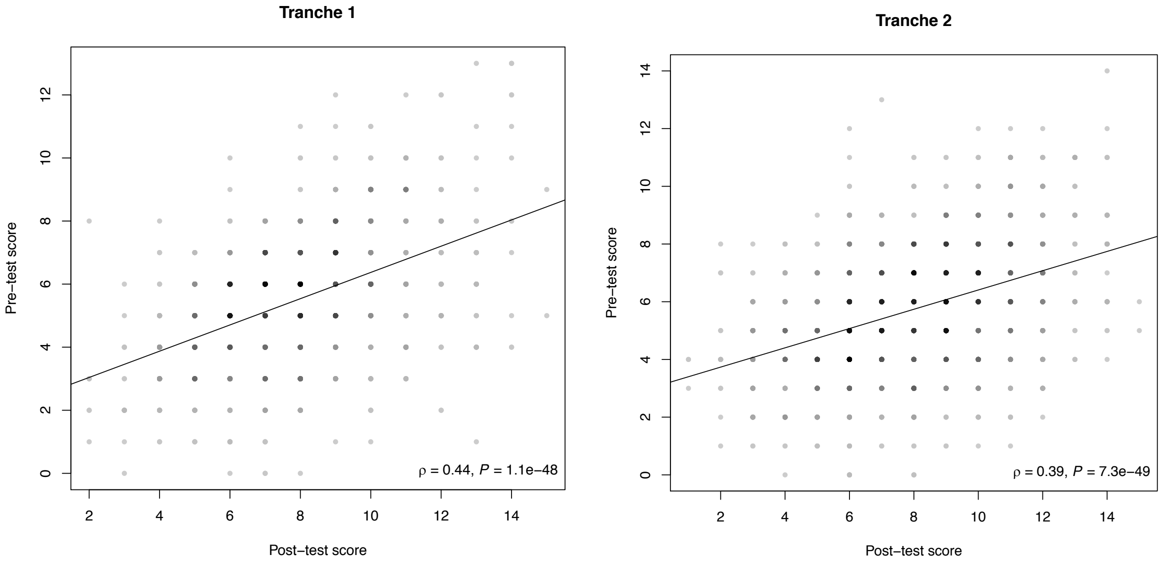

Supplementary Figure 2. Box plots showing the stratification of pre-test score by teacher assessed science ability for both tranches Note; tranche 1: high ability: mean = 6.42 ± 2.09, median = 6.00, middle ability: mean = 5.27 ± 1.20, median = 5.00, low ability: mean = 4.44 ± 1.09, median = 4.00; tranche 2: high ability: mean = 6.84 ± 2.18, median = 7.00, middle ability: mean = 5.55 ± 2.15, median = 5.50, low ability: mean = 4.73 ± 2.10, median = 5.00.

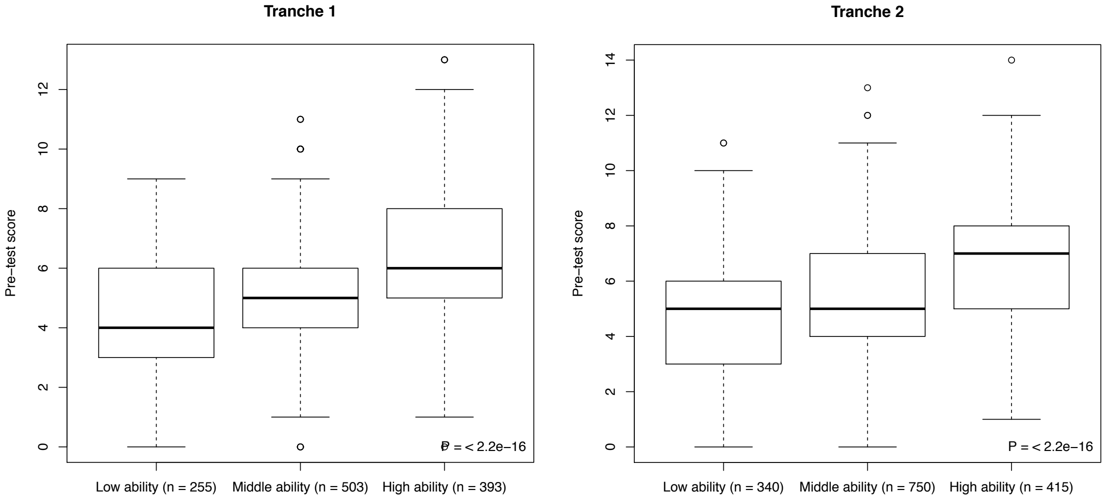

Supplementary Figure 3: Box plots comparing matched pre, post-teaching and retention scores for both tranches of data. Note; tranche 1: n = 320, pre-test: mean = 5.50 ± 2.16, median = 5.00, post-test: mean = 7.90 ± 2.26, median = 8.00, retention test: mean = 7.34 ± 2.52, median = 7.00; tranche 2: n = 523, pre-test: mean = 5.91 ± 2.24, median = 6.00, post-test: mean = 8.19 ± 2.86, median = 8.00, retention test: mean = 8.03 ± 2.92, median = 8.00.

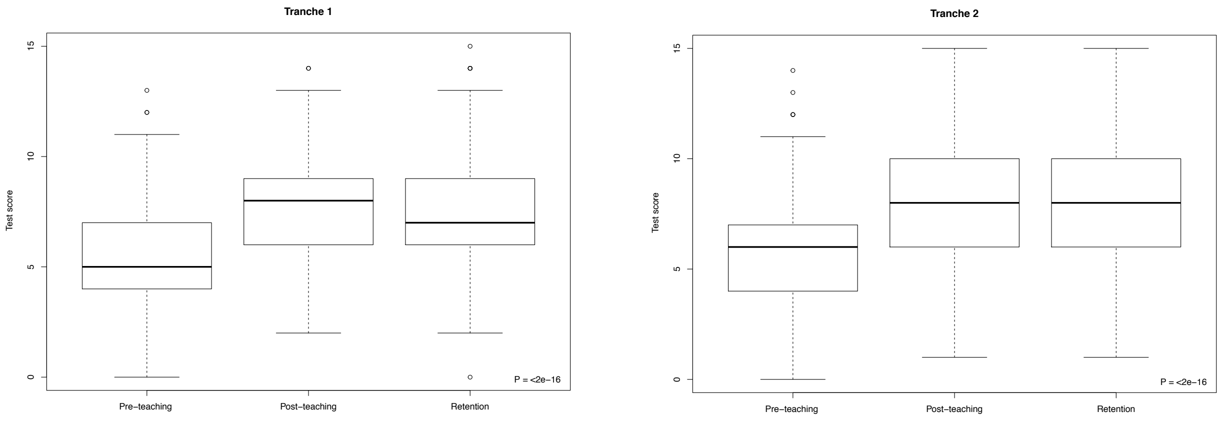

Supplementary Figure 4. Scatterplots with lines of regression showing correlation between MATE and CINS scores for participating teachers for both tranches (tranche 1: N = 27; tranche 2 N = 43).

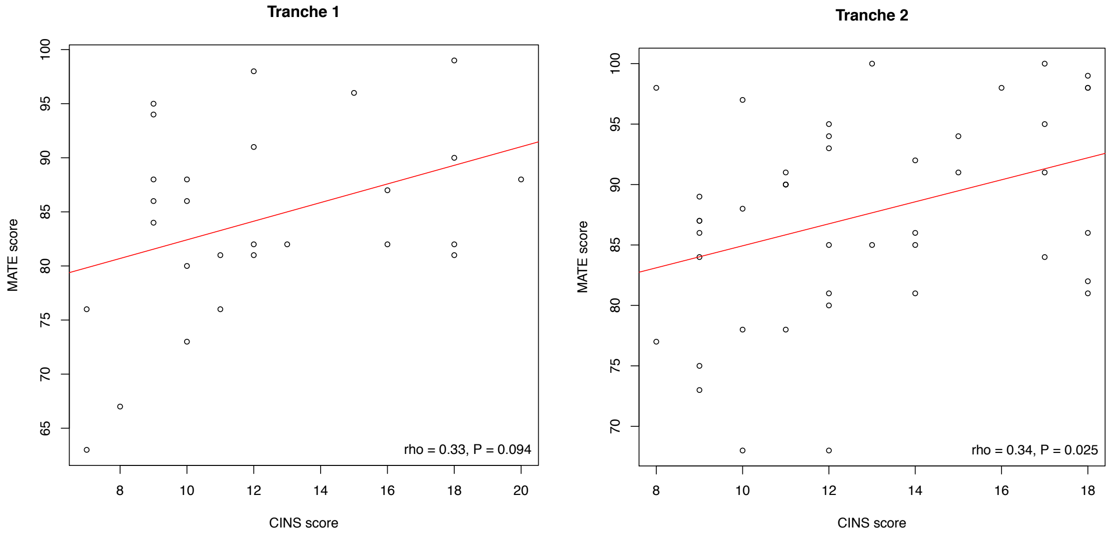

The mean MATE scores for teachers in both tranches (tranche 1: 84.30 ± 8.71; tranche 2: 87.45 ± 8.30) reflects a high acceptance of evolution (high acceptance MATE > 77 [13]), there being no significant difference between MATE scores in the two tranches (P = 0.14, Wilcoxon rank-sum test) with a small effect size (Cliff's d = 0.21, 95% CI: -0.41 – 0.01).
